# NLRP12 decreases TRIM25-mediated HK2 degradation to promote glycolysis and H3K18la in gastric cancer

**DOI:** 10.1038/s41419-025-07923-3

**Published:** 2025-08-13

**Authors:** Linsen Zhou, Zhiqiang Wang, Yang Huang, Xinyi Zhang, Haohai Jiang, Zhiyuan Guo, Guangjun Zhou, Haofeng Liu

**Affiliations:** 1 Department of General Surgery, The Yancheng Clinical College of Xuzhou Medical University & The First People’s Hospital of Yancheng, Yancheng, China; 2 Department of General Surgery, Tumor Hospital Affiliated to Nantong University & Nantong Tumor Hospital, Nantong, China; 3 Graduate School of Dalian Medical University, Dalian, China; 4 Nanjing Medical University Affiliated Changzhou Second People’s Hospital, Changzhou, China; 5 Department of Gastroenterology, The Yancheng Clinical College of Xuzhou Medical University & The First People’s Hospital of Yancheng, Yancheng, China

**Keywords:** Gastric cancer, Tumour biomarkers, Glycobiology, Ubiquitylation, Epigenetics

## Abstract

Gastric cancer is the most common primary malignant tumor of the digestive system. Recent studies have shown that targeting tumor cell metabolic reprogramming is a key cancer treatment strategy. NLR family pyrin domain containing 12 (NLRP12) is related to innate immunity, inflammation and tumorigenesis, but its role in the progression of gastric cancer remains unclear. The present study revealed that NLRP12 was highly expressed in gastric cancer tissues and cells, as well as positively correlated with poor patient prognosis and survival. NLRP12 promoted the progression of gastric cancer mainly by promoting the metabolic reprogramming of gastric cancer cells, the expression of histone H3 lysine 18 lactylation (H3K18la) and the stabilization of hexokinase 2 (HK2), a crucial enzyme in glycolysis. In the present study, NLRP12 competes with HK2 for binding to TRIM25, selectively reducing the K63-linked ubiquitination of HK2. Moreover, NLRP12 also exerted a significant cancer-promoting effect in mouse models. In summary, the present study demonstrated that NLRP12 prevents TRIM25 from mediating the K63-linked ubiquitination of HK2, which inhibits HK2 degradation through the autophagosome-lysosome pathway, thereby increasing its protein stability. These changes increase lactic acid production and induce H3K18la, which increases Myc transcription, thereby advancing gastric cancer progression. These findings reveal a novel cancer-promoting mechanism of NLRP12, potentially leading to the identification of new therapeutic targets for gastric cancer treatment.

## Introduction

Gastric cancer is one of the most common primary digestive system malignancies in adults. Due to its hidden early symptoms and often late detection, the current therapeutic strategies for gastric cancer are not effective [1, 2]. Gastric cancer cells often exhibit heterogeneous glycolysis and lactate metabolism, and previous studies have shown that glycolysis inhibition in gastric cancer cells selectively triggers histone lactylation [3, 4]. Therefore, the development of novel diagnostic prognostic indicators for gastric cancer and the study of the mechanisms of glucose metabolism and lactic acid metabolism in gastric cancer will help to identify key biomarkers and formulate effective treatment strategies [5].

Gastric cancer cells rely on various metabolic sources, and the specific nutrients they use are influenced by both cancer cell genetics and environmental conditions [3]. Gastric cancer cells often convert glucose to lactic acid even in the presence of oxygen, a process called aerobic glycolysis or the Warburg effect [6]. Hexokinase 2 (HK2) is a key metabolic enzyme that catalyzes the first reaction in the glycolytic pathway, phosphorylating glucose to glucose 6-phosphate [7]. Previous studies have shown that gastric cancer cells have high glycolytic activity and high HK2 expression [8]. The downstream components of HK2 are further excited by phosphofructokinase and pyruvate kinase, finally generating lactic acid under the catalytic action of lactate dehydrogenase to promote histone lactylation [9]. Histone lactylation, as a post-translational modification of histones, plays a role in gene transcriptional regulation. Previous studies have shown that H3K18la-mediated VCAM1 expression promotes gastric cancer progression and metastasis via the AKT‒mTOR‒CXCL1 axis [10]; lactic acid accumulation resulting in H3K18la promotes PD-L1 transcription, thereby mediating the immune escape of gastric cancer cells [11]. This phenomenon is due to the heterogeneity of glucose metabolism and lactic acid metabolism in gastric cancer cells, which provides new ideas for the treatment of gastric cancer.

During the development of gastric cancer, various aberrations occur in the protein degradation pathway, including those in the ubiquitin–proteasome pathway and the autophagy–lysosome pathway [12]. Among these modifications, the ubiquitin (Ub) modification is the most extensive, abundant and conserved eukaryotic internal translational modification [13]. Ubiquitination is a process in which ubiquitin is covalently bound to the target protein under the catalysis of a series of enzymes, mainly by the synergistic interaction of the E1 ubiquitin activator enzyme, the E2 ubiquitin coupling enzyme and the E3 ubiquitin ligase [14]. Tripartite motif 25 (TRIM25) is part of a large class of proteins with E3 ubiquitin ligase activity involved in different cellular functions [15]. Previous studies have shown that TRIM25 promotes the survival and growth of hepatocellular carcinoma cells through targeting the Keap1‒Nrf2 pathway [16]; Trim25 inhibits ITPKB degradation and imparts TMZ resistance in glioblastoma through ROS homeostasis [17]. Glucose and lactic acid metabolic reprogramming in gastric cancer cells largely depends on the protein stability of HK2 [7]. A previous study on post-translational modifications has reported that HK2 has many lysine sites that allow ubiquitination, and the ubiquitination of HK2 has been reported to occur in some tumor cells [18]. However, the TRIM25-mediated ubiquitination of HK2 has been less studied, especially in gastric cancer. These studies suggest that targeting HK2 protein stability by interfering with ubiquitination is an effective strategy for interfering with the metabolic reprogramming of gastric cancer cells.

NOD-like receptor family pyrin domain containing 12 (NLRP12) is one of the main members of the NOD-like receptor (NLR) family of innate immune system pattern recognition receptors [19]. NLRP12 has been shown to regulate inflammatory responses, immune cell infiltration and microbial interactions, thereby driving tumorigenesis [20]. NLRP12 has also been reported to play a functional regulatory role in various cancers, including colorectal cancer [21], liver cancer [22], and prostate cancer [23], and it is associated with patient prognosis. However, the role of NLRP12 in gastric cancer has not been well studied. Previous studies have shown that NLRP12 downregulates the Wnt/β-catenin pathway by interacting with STK38 to inhibit colorectal cancer [21]. In addition, NLRP12 has been reported to regulate RIG-I ubiquitination degradation through its interaction with TRIM25 [24]. By screening the TCGA database, analyzing the GSE134520 single-cell sequencing data, and verifying via clinical data collection, the present study revealed that NLRP12 was highly expressed in gastric cancer cells and was positively correlated with poor patient prognosis. NLRP12 is a molecular target highly correlated with the diagnosis, treatment, and prognosis of gastric cancer. Overall, targeting NLRP12 for tumor treatment has considerable potential, but the specific mechanism of action involving NLRP12 needs to be further explored and studied.

Therefore, we employed NLRP12 knockdown and overexpression cellular models along with xenograft mouse models to investigate the pivotal role and characteristics of NLRP12 in gastric cancer progression. We specifically focused on examining NLRP12’s inhibitory effect on TRIM25-mediated K63-linked ubiquitination of HK2. The present findings will help to explore new gastric cancer biomarkers and prognostic indicators, elucidate the mechanism of glucose and lactic acid metabolic reprogramming during gastric cancer progression, and lay a foundation for the clinical treatment of gastric cancer.

## Materials and methods

### Database analysis

Differential expression of NLRP12 in gastric cancer and adjacent noncancerous tissues was analyzed using The Cancer Genome Atlas (TCGA, https://cancergenome.nih.gov/), and Kaplan‒Meier survival prediction analysis was performed.

### Human gastric cancer tissues

Gastric cancer tissue and paracancerous normal tissue (*n* = 68) were obtained from the General Surgery Sample Bank of Yancheng Clinical College, Yancheng Clinical College, Xuzhou Medical University, from June 30, 2022, to June 30, 2024. All patients provided informed consent, and the clinical information related to the human samples is shown in Table 1. The samples were collected immediately after surgical resection, rapidly frozen in liquid nitrogen and stored at −80 °C. This study was approved by the Ethics Committee of Yancheng Clinical College, Xuzhou Medical University (2024-K(YJ)-183).

**Table 1:** Correlation between NLRP12 expression and clinicopathologic characteristics of gastric cancer patients.

Characteristics	Number	NLRP12 Low	NLRP12 High	*P*-value
All patients	68	28	40	
Gender				0.772
Male	33	13	20	
Female	35	15	20	
Age at diagnosis				0.414
<60	30	14	16	
≥60	38	14	24	
Clinical stage				0.006**
I	9	7	2	
II	16	10	6	
III	20	6	14	
IV	23	5	18	
Metastasis				0.020*
No	45	23	22	
Yes	23	5	18	
Vascular invasion				0.368
No	32	15	17	
Yes	36	13	23	

*P < 0.05, **P < 0.01.

### Cell culture and reagents

Human gastric mucosal epithelial cell lines (GES-1) and human gastric cancer cell lines (SNU-216, AGS, HGC-27, Ncl-N87, and MKN-45) were purchased from the United States Type Culture Bank (ATCC). All the cells were cultured in Dulbecco’s modified Eagle’s medium (DMEM; Gibco, USA) supplemented with 10% Fetal Bovine Serum (FBS; Gibco, USA) or Roswell Park Memorial Institute medium 1640 (RPMI-1640; Gibco, USA) supplemented with 10% Fetal Bovine Serum (FBS; Umedium, He fei, China), 100 µg/mL streptomycin and 100 U/mL penicillin (Gibco, USA). The cells were cultured in an incubator at 37 °C and 5% CO_2_. All the cell lines were identified by short tandem repeat (STR) analysis. The 2-DG (MedChemExpress, MCE, USA) was dissolved in dimethyl sulfoxide (DMSO; Servicebio, China).

### Cell Counting Kit-8 (CCK-8) assay

Cell viability was assessed by a CCK-8 assay (Abbkine, China). AGS and MKN-45 gastric cancer cells were inoculated into 96-well plates at a density of 3000 cells/well and incubated at 37 °C for 0, 24, 48 and 72 h. Then, 10 µL of CCK-8 reagent was added to each well, and the plates were incubated at the same temperature for 2 h. Absorbance was measured at 450 nm using a multifunctional microporous plate apparatus (Thermo Fisher Scientific, USA).

### Colony formation assay

The colony formation rate is an indicator of cell proliferation ability. The cells were inoculated in 6-well plates at a density of 400 cells/well and cultured in DMEM for 14 days. After the colonies were directly observed, they were fixed with 4% paraformaldehyde and stained with crystal violet. The colony numbers were quantified using ImageJ.

### 5‑Ethynyl‑2ʹ‑deoxyuridine (EdU) proliferation assay

Cell proliferation was quantified via the EdU proliferation method. The cells were inoculated into 96-well plates at a density of 3 × 10^3^ cells/well and incubated for 24 h. After incubation with EdU solution (10 μM, Abbkine, China) for 2 h, the cells were fixed with a solution containing 4% paraformaldehyde and then permeabilized with PBS containing 0.5% Triton X-100 for 10 min. The cells were incubated with the corresponding staining solution for approximately 30 min, and DAPI was used to stain the nuclei. Observations were made using an Olympus microscope (Tokyo, Japan). DAPI stained the nuclei blue, and the proliferating cells emitted red or green fluorescence. On the basis of these observations, the proportion of proliferating cells was determined. The total cell counts and proliferative cell counts were quantitatively analyzed by ImageJ.

### Seahorse analyzer

The extracellular acidification rate (ECAR) was measured with an XF24 extracellular flux analyzer (Agilent Technologies, USA). In brief, 1 × 10^5^ cells in 250 μL of DMEM were inoculated into each well of a Seahorse XF 24 plate and incubated overnight. The ECAR values were normalized to the number of cells. The ECAR of the cells in XF hippocampal culture medium containing 2 mM glutamine was measured after injection of 2 µM oligomycin, 100 mM 2-DG, and 30 mM glucose.

### Measurement of lactic acid levels

Lactic acid levels were determined with a lactic acid assay kit (Abbkine, Wuhan, China). In brief, lactate reacts with the enzyme mixture to produce the product, which is then added to the lactic acid probe, and the absorbance is read at 570 nm via a microplate reader (Thermo Fisher, Massachusetts, USA).

### Western blot analysis

Total protein was extracted using a radioimmunoprecipitation buffer (RIPA, Servicebio, China) supplemented with a protease inhibitor (Beyotime, China). The protein concentration was determined using a bicinchoninic acid (BCA) kit (Thermo Fisher Scientific, USA). The same amount of protein was then loaded onto a sodium dodecyl sulfate (SDS)-polyacrylamide gel and transferred to a polyvinylidene fluoride (PVDF) membrane (Millipore, USA). After incubation with the primary antibody at 4 °C overnight, the membrane was incubated with a horseradish peroxidase (HRP)-conjugated secondary antibody. After incubation with an enhanced chemiluminescence (ECL) solution (Abbkine, China), the protein bands were visualized via a ChemiDoc Imaging System (Bio-Rad, USA). Density quantification was performed, and β-actin was used as a control. The following primary antibodies were used: anti-NLRP12 (30998-1-AP, Proteintech, China), anti-pan-Kla (Jingjie, PTM-1401RM, China), anti-H3K18la (Jingjie, PTM-1406RM, China), anti-HK1 (#2024 S, CST, USA), anti-HK2 (#2867 S, CST, USA), anti-PFKL (#ER64567, HUABIO, China), anti-PFKM (# ET7106-97, HUABIO, China), PFKP (#HA500472, HUABIO, China), anti-PKM1 (#7067 S, CST, USA), anti-PKM2 (#4053 S, CST, USA), anti-LDHA (#3582 S, CST, USA), anti-LDHB (#0807-1, HUABIO, China), anti-TRIM25 (12573-1-AP, Proteintech, China), anti-LAMP2 (66301-1-Ig, Proteintech, China), Ubiquitin (P23347, Promab, China), anti-histone H3 (A2348, ABclonal, China) and anti-β-actin (AC026, ABclonal, China).

### Quantitative reverse transcription‒PCR (RT‒qPCR)

Total RNA was isolated from cells or tissues with an RNA-Easy separation reagent (Vazyme, China). The same amount of RNA was reverse transcribed into cDNA using HiScript II Q RT SuperMix for qPCR (+gDNA Wiper) (Vazyme, China). ChamQ Universal SYBR qPCR Master Mix (Vazyme, China) was mixed with cDNA and gene-specific primers, and qPCR was performed according to the manufacturer’s protocol. The mRNA level of the target gene was normalized to the corresponding β-actin level via the 2^−^^∆∆Ct^ method. The following primers were used: NLRP12 forward, ACCAGACCTTGACCGACCTT; NLRP12 reverse, GAGGACTCGGAGTTTGCAGC; HK2 forward, 5’-GAGCCACCACTCACCCTACT-3’; HK2 reverse, 5’-CCAGGCATTCGGCAATGTG-3’; TRIM25 forward, 5’-AGGGATGAGTTCGAGTTTCTGG-3’; TRIM25 reverse, 5’-GTTTTTGAGGTCTATGGTGCTCT-3’, β-actin forward, 5’-CTCCATCCTGGCCTCGCTGT-3’; and β-actin reverse, 5’-GCTGTCACCTTCACC GTTCC-3’.

### ChIP assay and ChIP‒qPCR

Gastric cancer cells were collected and fixed in PBS with 1% formaldehyde for 10 min. To stop the crosslinking process, 1.25 mol/L glycine was added. After the washing step, the fixed cells were resuspended in lysis buffer (1% SDS; 50 mmol/L Tris-HCl, pH 8.1; 5 mmol/L EDTA; and protease inhibitor) for 1 h. The cells were ultrasonicated and centrifuged at 4000 rpm at 4 °C for 10 min. The lysate was diluted in buffer containing 1% Triton X-100, 20 mmol/L Tris-HCl (pH 8.1), 150 mmol/L NaCl, 2 mmol/L EDTA, and a protease inhibitor. Rabbit anti-IgG (2 µg) or rabbit anti-H3K18La (2 µg) was added to the diluted chromatin, which was incubated overnight with constant rotation at 4 °C. The next day, 30 µL of Dynabeads™ protein G (Invitrogen, USA) was added, and the mixture was incubated at 4 °C for 4 h. Following the washing procedure, the input and pull-down chromatin complexes were decrosslinked at 65 °C for 12 h. The DNA was then purified via a MinElute PCR purification kit (Qiagen, Hilden, Germany). ChIP‒qPCR was performed with PerfectStart Green qPCR SuperMix (TransGen Biotech, Beijing, China) on a QuantStudio5 real-time fluorescence quantitative PCR system [25]. The following Myc primers were used: forward, TTCTGAAACCTGGCTGAGAAA; reverse, CTAGGGCGAGAGGGAGGTT.

### Protein and RNA stability assays

Gastric cancer cells transfected with shRNA were seeded into a 6-well plate. The cells were subsequently treated with 100 μg/mL cycloheximide (CHX) or 5 μg/mL actinomycin D (Actd) when the cells reached 70% confluence [26]. The cells were collected at a specified time point, after which Western blot analysis and RT‒qPCR were performed.

### Co-immunoprecipitation (Co-IP) assay

For verification of protein interactions, the cells were lysed with NP-40 cell lysis buffer (Beyotime, China) supplemented with protease inhibitors. After centrifugation, superalbumin was isolated via SDS‒PAGE and evaluated by Western blot analysis. The cell lysate was mixed with an anti-NLRP12 antibody, anti-TRIM25 antibody (4 μg/mL) or control IgG (AC011, ABclonal, China) and then mixed with protein A/G beads (20241, Invitrogen, USA), followed by incubation at 4 °C for 3 h. The samples were carefully washed with precooled PBS, mixed with protein-loading buffer, boiled, and evaluated by Western blot analysis with an anti-NLRP12 antibody or anti-TRIM25 antibody. To analyze ubiquitination, 293T cells were cotransfected with different plasmids. The lysate was precipitated through repeated IP procedures, and the proteins were evaluated by WB analysis.

### LysoIP

Following two washes with ice-cold PBS, cells were harvested in 1 mL PBS supplemented with protease/phosphatase inhibitors and centrifuged (1000 × *g*, 2 min, 4 °C). Cell pellets were resuspended in 800 μL inhibitor-supplemented PBS, with 20 μL aliquoted as whole-cell controls. The remaining suspension was homogenized using a 2 mL Dounce homogenizer. After low-speed centrifugation (1000 × *g*, 2 min, 4 °C) to pellet debris/intact cells, the organelle-containing supernatant was subjected to immunoprecipitation with 10 μL anti-TMEM192 polyclonal antibody and 100 μL Protein A/G magnetic beads. The immunocomplex was washed thrice with PBS using magnetic separation, followed by protein extraction in 100 μL ice-cold 1% Triton X-100 lysis buffer. Beads were subsequently removed magnetically.

### Xenograft mouse model

All animal experiments were approved by the Animal Health Committee of Jiangsu Medical Vocational College (Approval No. XMLL-2024-800). To establish a subcutaneous xenograft model, 3 × 10^6^ empty vector gastric cancer cells, OE-NLRP12 gastric cancer cells or sh-HK2 gastric cancer cells were injected into the right backs of 6-week-old nude mice after inhalation anesthesia with isoflurane (*n* = 6 mice per group). The samples were grouped according to the needs of the experiment. The mice were monitored regularly for 4 weeks and then euthanized. After the experiment, the tumor tissue was surgically removed, fixed, and embedded for subsequent experiments.

### IHC staining

Immunohistochemical staining was used to evaluate protein expression in tumor tissues. Briefly, the tumor tissue was fixed with 4% paraformaldehyde and embedded in paraffin. The tumor tissue was then sectioned, decarbonized, hydrated, washed and incubated overnight at 4 °C with specific primary antibodies. The next day, the sections were washed and incubated with secondary antibodies. The immune response signal was detected by 3,3’-diaminobenzidine (DAB). The nuclei were reverse-stained with hematoxylin. The samples were then examined under a microscope.

### Plasmids, RNA interference, and transfection

Short hairpin RNA (shRNA) and wild-type plasmids were constructed by Nanjing Zebrafish Biotechnology Co., Ltd. Briefly, polymerase chain reaction (PCR)-amplified human HK2, TRIM25, and NLRP12 were cloned and inserted into the pLV-CMV-MCS-puro-3×Flag vector, pLV-CMV-MCS-puro-6×His or pLV-CMV-MCS-puro-myc vector. The shRNA target sequences were subsequently cloned and inserted into the PLKO.1-puro vector. HEK-293T cells were cotransfected with the recombinant plasmids and packaging plasmids using Polyplus according to the manufacturer’s instructions (Sartorius, Germany). At 24 to 48 h later, the lentiviral particles were collected and stored at −80 °C. For stable cell line construction, cells were infected with lentivirus in culture medium supplemented with 5 μg/mL polybrene (Beyotime Biotechnology, China) for 24 h. The cells were treated with puromycin (Biotechnology, China) for positive clone selection. The gene expression efficiency was determined by quantitative reverse transcription PCR (RT‒qPCR) or immunoblot analyses. The following shRNA sequences were used: shNLRP12-1, TGGAACTGAAGAAGTTCAAGTTA; shNLRP12-2, CTGGAATCAGAAAATCCTATTTG; shHK2, GGAGCTCAACCATGACCAAGT; and shTRIM21, GGGTCAACAGCAAGTTTGA.

### Statistical analysis

To compare continuous variables between two groups and among more than two groups, unpaired Student’s *t*-test and one-way or two-way ANOVA, respectively, were employed. The results are presented as the means, and the error bars indicate the standard deviations (mean ± SD). A Kaplan–Meier model was used to conduct survival analysis. Statistical analyses were performed via GraphPad Prism 8.0. Bioinformatics analyses were performed as previously described. The criteria for statistical significance were as follows: *P < 0.05; **P < 0.01; and ns, nonsignificant.

## Results

### NLRP12 is upregulated and associated with prognosis in gastric cancer

**Fig. 1:**
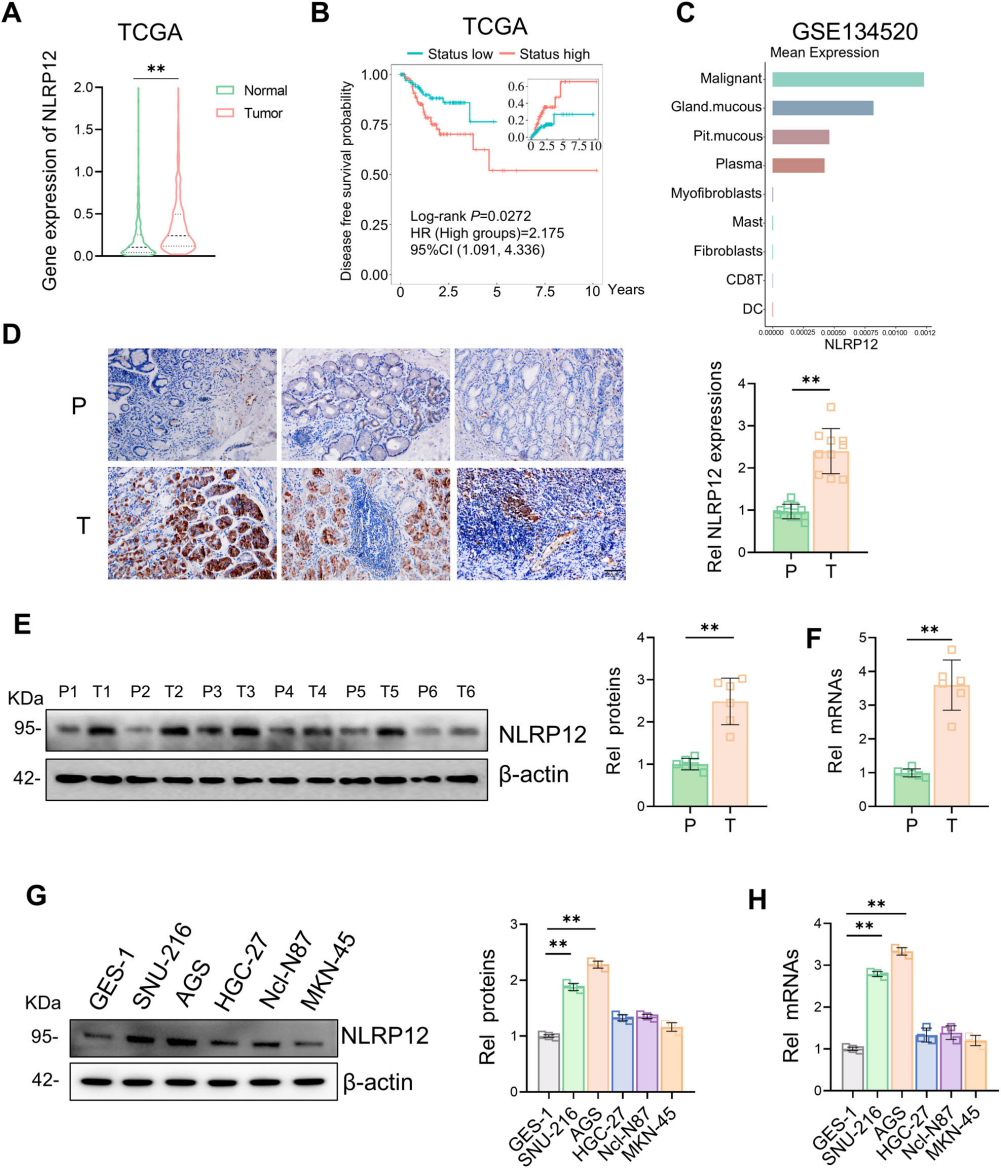
NLRP12 is upregulated and associated with prognosis in gastric cancer. **A** The expression of NLRP12 in gastric cancer and adjacent normal tissues was analyzed using TCGA database (**P < 0.01). **B** Disease-free survival was determined on the basis of NLRP12 expression in patients with clinically diagnosed gastric cancer using the TGGA database. **C** The GSE134520 dataset was used to analyze the distribution of NLRP12 in different gastric cancer cells. **D** Immunohistochemical staining of NLRP12 in gastric cancer and adjacent normal tissues and quantitative analysis (means ± SD, **P < 0.01). **E** Western blot analysis was used to detect NLRP12 protein expression levels in different gastric cancer tissues and adjacent normal tissues, and the results were quantitatively analyzed (means ± SD, **P < 0.01). **F** The expression of NLRP12 mRNA in gastric cancer and adjacent normal tissues was detected by RT-qPCR (means ± SD, **P < 0.01). **G** Western blot analysis was used to detect the protein expression of NLRP12 in GES-1, SNU-216, AGS, HGC-27, Ncl-N87 and MKN-45 cells, and the results were quantitatively analyzed (*n* = 3, means ± SD, *P < 0.05). **H** The differential expression of NLRP12 mRNA in GES-1, HGC, MGC, MKN, SGC and AGS cells was analyzed by RT-qPCR (*n* = 3, means ± SD, *P < 0.05).

To investigate the role of NLRP12 in gastric cancer progression, we used the TCGA database and the GSE134520 dataset [27] to analyze its expression in gastric cancer tissues and adjacent normal tissues. According to TCGA data analysis, NLRP12 was significantly overexpressed in gastric cancer tissues, and high NLRP12 expression was associated with shorter disease-free survival periods (Fig. 1A, B). The GSE134520 dataset involves single-cell sequencing analysis of gastric cancer, and analysis of this dataset revealed that NLRP12 was expressed mainly in malignant gastric cancer cells and also partially expressed in gland mucous, pit mucous and plasma (Fig. 1C). IHC analyses confirmed that NLRP12 was specifically upregulated in gastric cancer tissues compared to adjacent normal tissues, where its expression was significantly lower (Fig. 1D). After dividing gastric cancer patients at Yancheng Clinical College of Xuzhou Medical University into a high-NLRP12 expression group and a low-NLRP12 expression group, we analyzed the correlations between the expression level of NLRP12 and the clinicopathological parameters of gastric cancer patients. The TNM grade of the high-expression group was significantly higher than that of the low-expression group (Table 1). In addition, analysis of RNA and protein expression in gastric cancer and paracancerous tissues further revealed that NLRP12 was more highly expressed in gastric cancer than in paracancerous tissues (Fig. 1E, F). To further investigate the expression of NLRP12 in various gastric cancer cell lines, RNA and protein were extracted from normal gastric mucosa cells (GES-1) and five gastric cancer cell lines (SNU-216, AGS, HGC-27, Ncl-N87 and MKN-45). The expression levels of NLRP12 in SNU-216 and AGS cells were significantly greater than those in GES-1 cells, while no significant difference in NLRP12 expression was observed in the remaining three gastric cancer cell lines (HGC-27, Ncl-N87, and MKN-45) compared to GES-1 cells (Fig. 1G, H).

### NLRP12 promotes gastric cancer proliferation both in vivo and in vitro

**Fig. 2:**
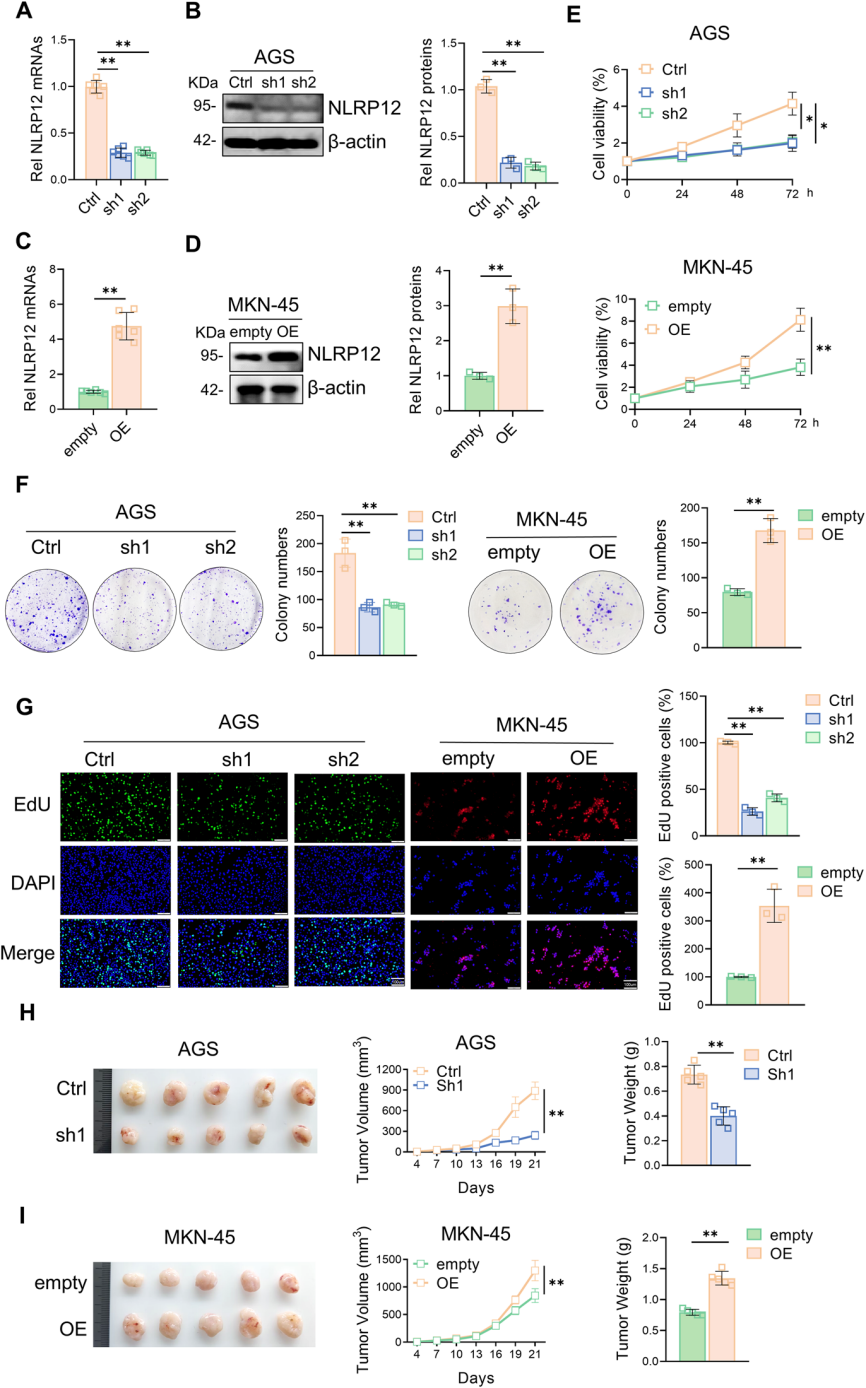
NLRP12 promotes gastric cancer proliferation both in vivo and in vitro. **A** After AGS cells were transfected with shRNA, RT‒qPCR was used to verify the transfection efficiency of sh-C5aR1 (*n* = 3, means ± SD, *P < 0.05). **B** After AGS cells were transfected with shRNA, the protein expression of NLRP12 was detected by Western blot analysis, and quantitative analysis was performed (*n* = 3, means ± SD, *P < 0.05). **C** After MKN-45 cells were transfected with the overexpression plasmid, RT‒qPCR was used to verify the transfection efficiency of OE-NLRP12 (*n* = 3, means ± SD, **P < 0.01). **D** After MKN-45 cells were transfected with the overexpression plasmid, the protein expression of NLRP12 was detected by Western blot analysis, and quantitative analysis was performed (*n* = 3, means ± SD, **P < 0.01). **E** A CCK-8 assay was used to evaluate the changes in gastric cancer cell viability after NLRP12 knockdown or overexpression (*n* = 3, means ± SD, *P < 0.05). **F** A colony formation assay was used to evaluate the changes in the proliferation ability of gastric cancer cells after NLRP12 knockdown or overexpression (*n* = 3, means ± SD, **P < 0.01). **G** An EdU assay was used to evaluate the changes in the proliferation ability of gastric cancer cells after NLRP12 knockdown or overexpression (*n* = 3, means ± SD, **P < 0.01). Scale bar, 100 μm. **H** Ctrl and sh-NLRP12 cells were injected subcutaneously into the flanks of BALB/c nude mice to observe the proliferation ability of the cells. Anatomical tumor images of BALB/c nude mice (*n* = 5). Tumor volume and tumor weight were assessed. **I** Empty vector and OE-NLRP12 cells were injected subcutaneously into the flanks of BALB/c nude mice to observe the proliferation ability of the cells. Anatomical tumor images of BALB/c nude mice (*n* = 5). Tumor volume and tumor weight were assessed.

To further explore the functional role of NLRP12 in gastric cancer progression, we knocked down NLRP12 in AGS cells and overexpressed NLRP12 in MKN-45 cells, and we verified the knockdown or overexpression efficiency by RT-qPCR and Western blot analysis (Fig. 2A–D). To verify the effect of NLRP12 on gastric cancer cells, a CCK-8 cell viability assay, colony formation assay and EdU staining assay were performed. Knockdown of NLRP12 significantly reduced the proliferation ability of gastric cancer cells, whereas overexpression of NLRP12 significantly enhanced the proliferation ability of gastric cancer cells (Fig. 2E–G). To further clarify the ability of NLRP12 to regulate the proliferation in gastric cancer cells, we constructed a tumor xenograft model in nude mice. Knockdown of NLRP12 significantly inhibited tumor size and volume in vivo, whereas the overexpression of NLRP12 increased tumor size and volume in vivo (Fig. 2H, I). In summary, NLRP12 regulates the proliferation of gastric cancer cells both in vivo and in vitro.

### NLRP12 promotes H3K18la and gastric cancer progression through glycometabolic reprogramming

**Fig. 3:**
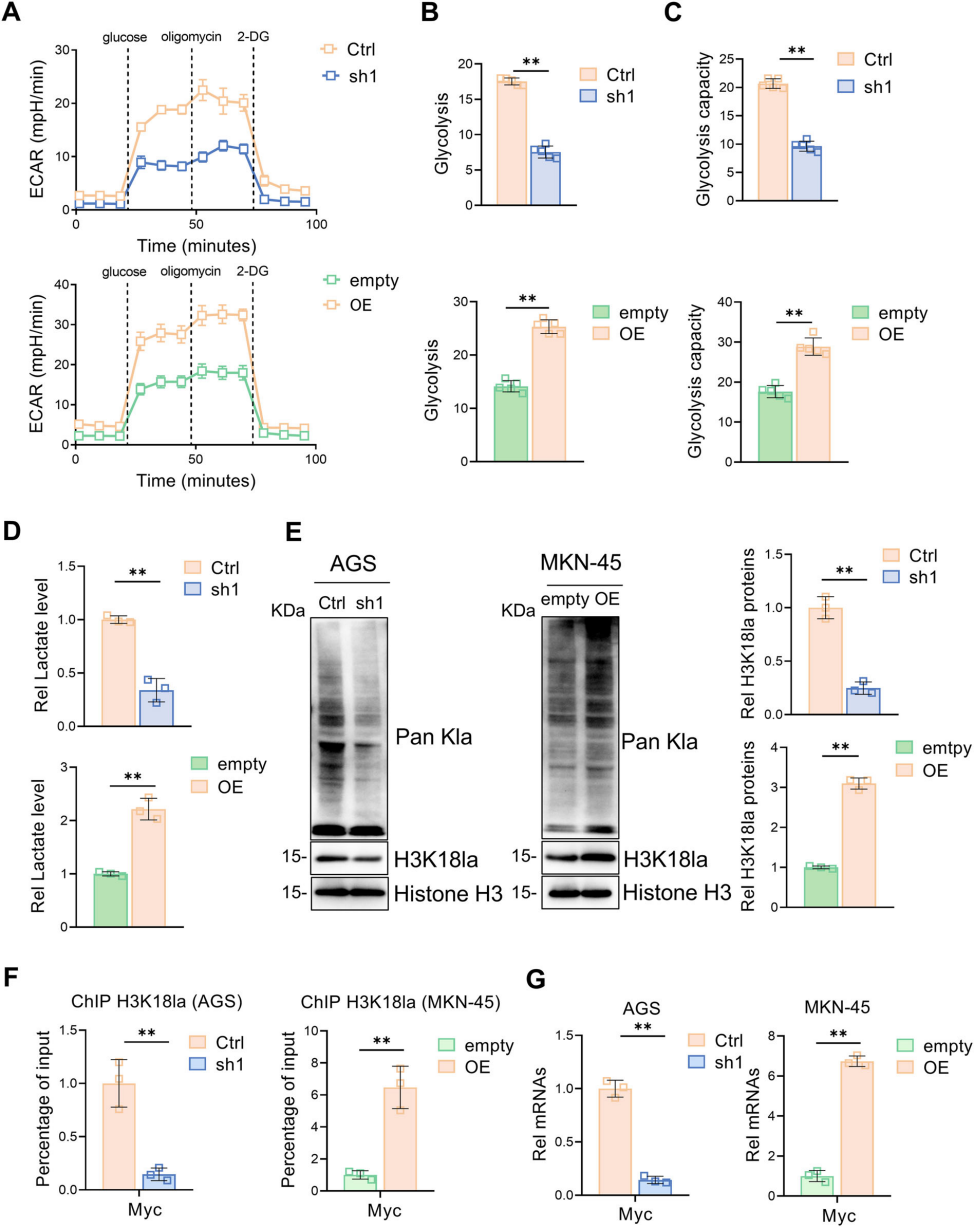
NLRP12 promotes H3K18la and gastric cancer progression through glycometabolic reprogramming. **A** A Seahorse assay was used to evaluate the changes in the extracellular acidification rate of gastric cancer cells after NLRP12 knockdown or overexpression. **B** The changes in glycolysis in gastric cancer cells after NLRP12 knockdown or overexpression were analyzed (*n* = 5, means ± SD, **P < 0.01). **C** The changes in the glycolytic capacity of gastric cancer cells after NLRP12 knockdown or overexpression were analyzed (*n* = 5, means ± SD, **P < 0.01). **D** The changes in lactate levels in gastric cancer cells after NLRP12 knockdown or overexpression were analyzed (*n* = 3, means ± SD, **P < 0.01). **E** Histone lactylation and H3K18la expression in gastric cancer cells after NLRP12 knockdown or overexpression were analyzed, and quantitative analysis was performed (*n* = 3, means ± SD, **P < 0.01). **F** The level of Myc binding to DNA in gastric cancer cells after NLRP12 knockdown or overexpression was analyzed by a ChIP assay (*n* = 3, means ± SD, **P < 0.01). **G** The Myc transcription level in gastric cancer cells after NLRP12 knockdown/overexpression was analyzed via RT‒qPCR (*n* = 3, means ± SD, **P < 0.01).

Previous studies have indicated that tumor cell growth relies heavily on the Warburg effect, which is characterized by increased glycolysis and lactic acid production [28]. To assess whether NLRP12 affects gastric cancer cell proliferation through glycolysis, we conducted Seahorse metabolic flux analysis. In gastric cancer cells, NLRP12 knockdown significantly decreased glycolysis levels and capacity, whereas NLRP12 overexpression significantly increased glycolysis levels and capacity (Fig. 3A–C). Lactic acid, the final product of glycolysis, was initially considered metabolic waste, but recent studies have shown that lactic acid modifies histone translation and plays a role in gene transcriptional regulation [29]. After knockdown of NLRP12, the overall lactate level and histone lactate level in gastric cancer cells decreased, whereas opposite effects were observed when NLRP12 was overexpressed. Through pan-lactylation screening and literature-based prioritization, we identified H3K18 as the most dynamically regulated lactylation site. Strikingly, NLRP12 knockdown reduced H3K18 lactylation levels, while NLRP12 overexpression robustly amplified this modification (Fig. 3D, E). Mechanistically, H3K18la has been reported to activate Myc transcription by enriching at its genomic locus [25]. and Myc-driven oncogenesis involves cell cycle progression, metabolic reprogramming, and ribosomal biogenesis [30]. Consistently, ChIP and RT-qPCR experiments revealed that the level of H3K18la and Myc transcription decreased after NLRP12 was knocked down, whereas the overexpression of NLRP12 increased the level of H3K18la and Myc transcription (Fig. 3F, G). To exclude off-target effects, rescue experiments were performed by overexpressing NLRP12 in shRNA-mediated knockdown cells, followed by validation through CCK-8 assays, Seahorse analysis, lactate quantification, and Pan-kla/H3K18la detection. The experimental results indicate that the changes in proliferation, glycolysis and downstream signals of gastric cancer cells are indeed caused by the changes in NLRP12 (Fig. S1). In conclusion, our findings demonstrate that NLRP12 drives gastric cancer cell proliferation via glycometabolic reprogramming, mechanistically linked to Myc transcriptional activation through H3K18la.

### NLRP12 promotes HK2-mediated gastric cancer H3K18la and proliferation

**Fig. 4:**
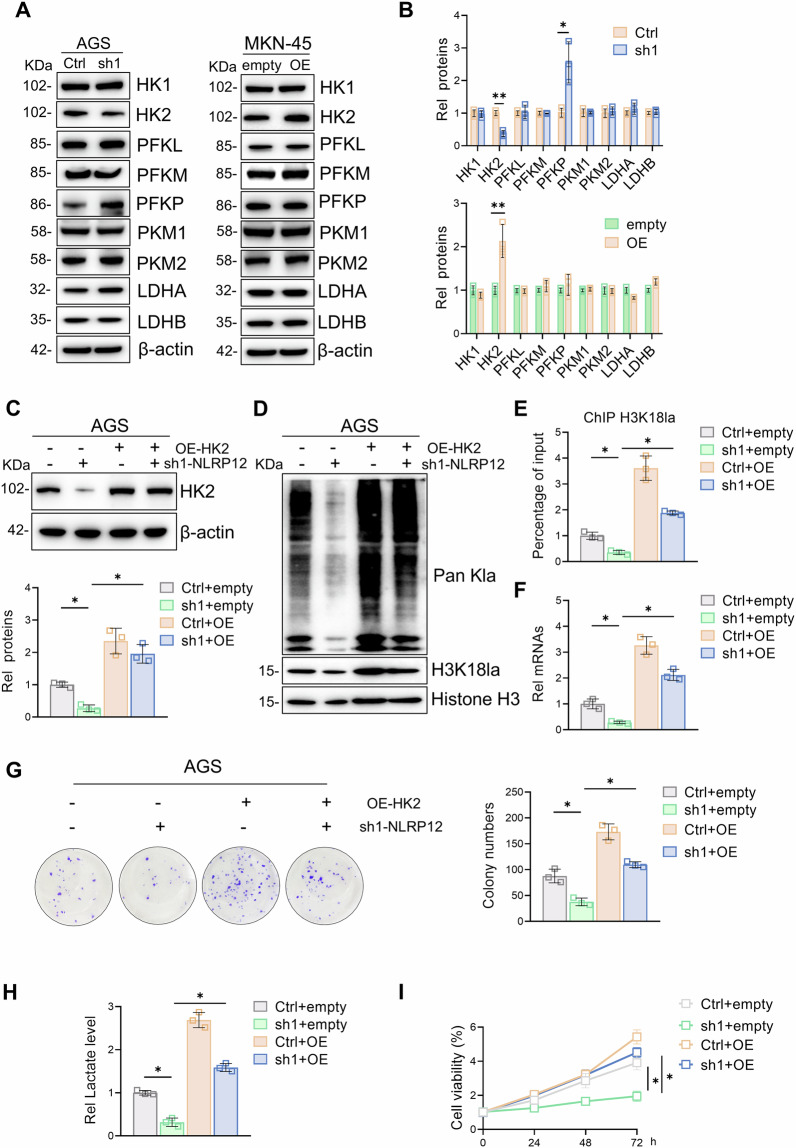
NLRP12 promotes HK2-mediated gastric cancer H3K18la and proliferation. **A** Western blot analysis was used to detect the expression of glycolysis-related enzymes. **B** Quantitative analysis of the data in A (*n* = 3, means ± SD, *P < 0.05 and **P < 0.01). **C** After overexpressing HK2 and knocking down NLRP12, the protein expression of HK2 was detected by Western blot analysis, and quantitative analysis was performed (*n* = 3, means ± SD, *P < 0.05). **D** After overexpressing HK2 and knocking down NLRP12, the expression of histone lactylation and H3K18la was detected by Western blot analysis. **E** After overexpressing HK2 and knocking down NLRP12, the transcription level of Myc-binding H3K18la was detected by ChIP (*n* = 3, means ± SD, *P < 0.05). **F** After overexpressing HK2 and knocking down NLRP12, the transcription level of Myc was detected by RT-qPCR (*n* = 3, means ± SD, *P < 0.05). **G** After overexpressing HK2 plasmid and knocking down NLRP12, a colony formation assay was used to detect the proliferation ability of gastric cancer cells, and quantitative analysis was performed (*n* = 3, means ± SD, *P < 0.05). **H** Lactate levels in gastric cancer cells were detected after overexpression of HK2 and knocking down NLRP12 (*n* = 3, means ± SD, *P < 0.05). **I** After overexpressing HK2 and knocking down NLRP12, the proliferation ability of gastric cancer cells was detected by a CCK-8 assay (*n* = 3, means ± SD, *P < 0.05).

To evaluate the specific mechanisms by which NLRP12 regulates glycolysis and lactate production in gastric cancer cells, we investigated the enzymes involved in glycolysis. Upon knockdown of NLRP12, HK2 expression is markedly downregulated, while PFKP protein levels are significantly upregulated. Conversely, on NLRP12 overexpression, the HK2 protein levels in gastric cancer cells significantly increased, whereas other glycolytic enzymes did not significantly change. Combining the results of the NLRP12 knockdown and overexpression experiments, we concluded that NLRP12 affects mainly the alteration of glycolysis by regulating the expression of HK2 (Fig. 4A, B). Furthermore, to establish causality, we performed rescue experiments by co-expressing HK2 in NLRP12-depleted cells, which restored lactate production, H3K18la, Myc transcription, and proliferation capacity (Fig. 4C–I).

### Targeting HK2 inhibits the NLRP12-mediated proliferation and progression of gastric cancer

**Fig. 5:**
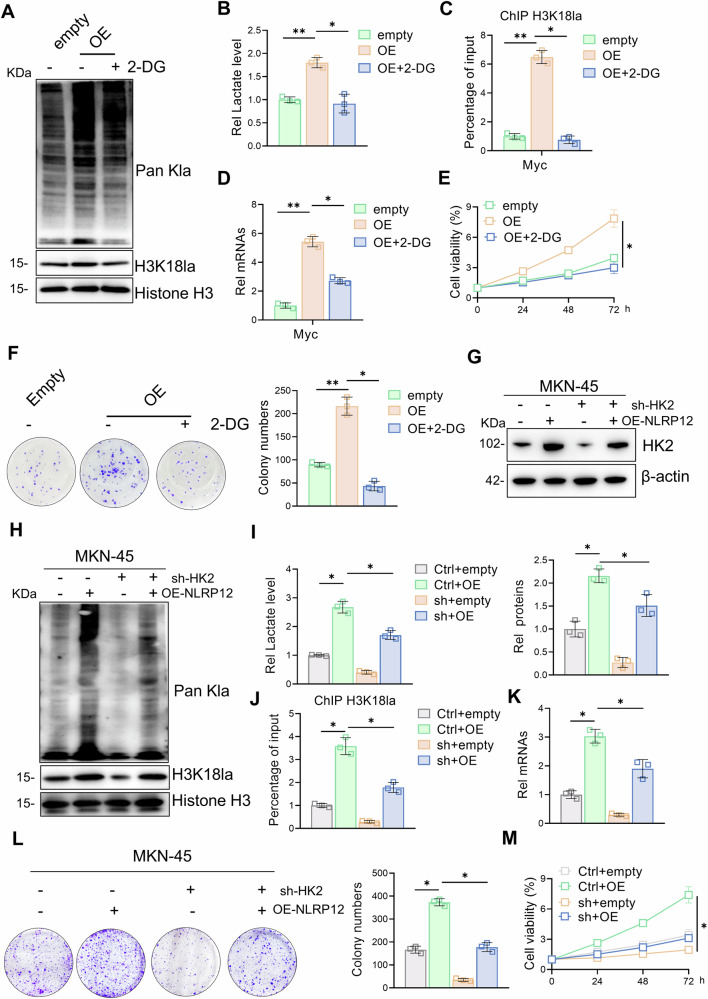
Targeting HK2 inhibits the NLRP12-mediated proliferation and progression of gastric cancer. **A** Histone lactylation and H3K18la levels were detected by Western blot analysis after treating NLRP12-overexpressing gastric cancer cells with 2-DG. **B** Lactate levels were detected after treating NLRP12-overxpressing gastric cancer cells with 2-DG (*n* = 3, means ± SD, *P < 0.05, **P < 0.01). **C** H3k18la-bound Myc levels were detected by a ChIP assay after treating NLRP12-overexpressing gastric cancer cells with 2-DG (*n* = 3, means ± SD, *P < 0.05, **P < 0.01). **D** Myc transcription levels were detected via RT‒qPCR after treating NLRP12-overexpressing gastric cancer cells with 2-DG. **E** Cell viability was detected by a CCK-8 assay after treating NLRP12-overexpressing gastric cancer cells with 2-DG (*n* = 3, means ± SD, *P < 0.05). **F** Cell viability was detected by a colony formation assay after NLRP12-overexpressing gastric cancer cells were treated with 2-DG (*n* = 3, means ± SD, *P < 0.05, **P < 0.01). **G** HK2 protein levels were detected by Western blot analysis after cotransfection of gastric cancer cells with the sh-HK2 and OE-NLRP12 plasmids, and quantitative analysis was performed (*n* = 3, means ± SD, *P < 0.05). **H** Histone lactylation and H3K18la levels were detected by Western blot analysis after cotransfection of gastric cancer cells with the sh-HK2 and OE-NLRP12 plasmids. **I** Lactate levels were detected after cotransfection of gastric cancer cells with the sh-HK2 and OE-NLRP12 plasmids (*n* = 3, means ± SD, *P < 0.05). **J** H3K18la-bound Myc levels were detected by a ChIP assay after cotransfection of gastric cancer cells with the sh-HK2 and OE-NLRP12 plasmids (*n* = 3, means ± SD, *P < 0.05). **K** Myc transcription levels were detected by RT‒qPCR after cotransfection of gastric cancer cells with the sh-HK2 and OE-NLRP12 plasmids (*n* = 3, means ± SD, *P < 0.05). **L** Cell viability was detected by a colony formation assay after cotransfection of gastric cancer cells with the sh-HK2 and OE-NLRP12 plasmids, and quantitative analysis was performed (*n* = 3, means ± SD, *P < 0.05). **M** Gastric cancer cell viability was determined by a CCK-8 assay after cotransfection of sh-HK2 and OE-NLRP12 plasmids (*n* = 3, means ± SD, *P < 0.05).

HK2 is a key metabolic enzyme that catalyzes the first step in the glycolysis pathway and phosphorylates glucose to glucose 6-phosphate, which plays an important role in promoting the Warburg effect [31]. To further clarify that NLRP12 promotes gastric cancer proliferation through HK2-mediated glycolysis, we employed both pharmacological inhibition (2-DG, a competitive HK2 inhibitor) and genetic silencing (shRNA-mediated HK2 knockdown). In NLRP12-overexpressing cells, 2-DG treatment significantly attenuated pan-Kla, H3K18la modification, lactate production, Myc transcription levels, and proliferative capacity (Fig. 5A–F). Moreover, HK2 depletion in the same cellular context phenocopied 2-DG-induced suppression (Fig. 5G–M). In summary, these data indicate that targeting HK2 disrupts NLRP12-mediated metabolic reprogramming and malignant progression in gastric cancer.

### NLRP12 stabilizes HK2 by blocking K63-linked ubiquitination and lysosomal targeting

**Fig. 6:**
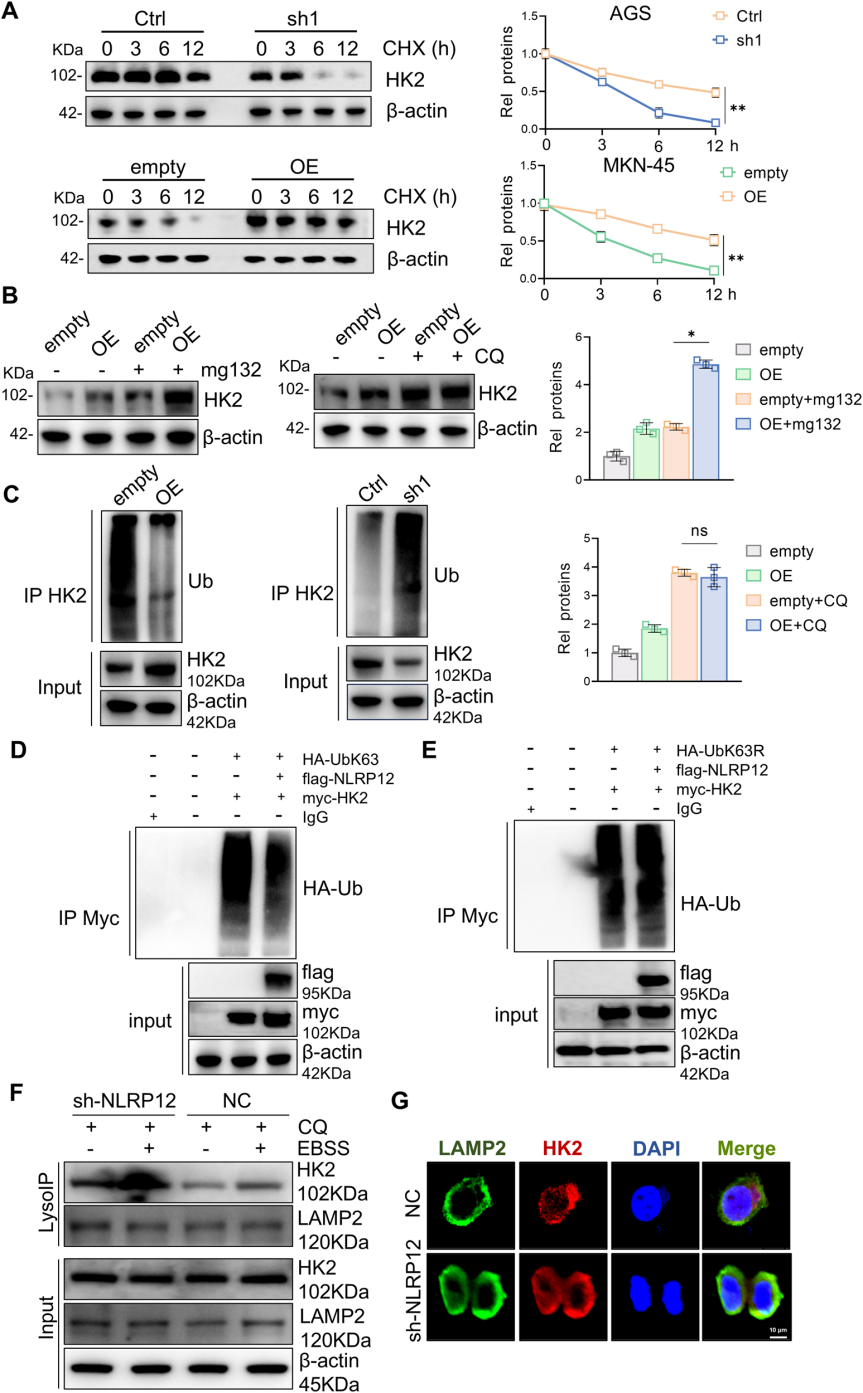
NLRP12 inhibits the degradation of HK2 through the Ub K63-linked mediated lysosomal pathway. **A** The protein stability of HK2 was examined after CHX treatment and after the knockdown or overexpression of NLRP12 (*n* = 3, means ± SD, **P < 0.01). **B** The protein level of HK2 was detected after treating NLRP12-overexpressing cells with MG132 (10 μM) or CQ (50 μM) for 6 h, and quantitative analysis was performed (*n* = 3, means ± SD, *P < 0.05). **C** The HK2 ubiquitination levels after NLRP12 knockdown or overexpression were detected by Western blot analysis. **D** The level of exogenous HK2 ubiquitination in 293T cells was detected by Western blot analysis. **E** HK2 ubiquitination levels in 293T cells after mutation of the K63-linked polyubiquitination were detected by Western blot analysis. **F** The content of HK2 protein in lysosomes was detected by lysosomal IP assay after CQ (50 μM) and EBSS treatment for 6 h. **G** HK2 and lysosomes were localized through the IF experiment after CQ (50 μM) and EBSS treatment for 6 h.

To further explore the specific mechanisms by which NLRP12 regulates HK2, we examined the transcription levels of all glycolytic enzymes. Neither the knockdown nor the overexpression of NLRP12 significantly altered HK2 transcription levels (Fig. S2A). To determine HK2 transcription and protein stability, gastric cancer cells were treated with CHX or ActD to block protein or mRNA synthesis, and HK2 degradation was evaluated over time (0, 3, 6 and 12 h) in NLRP12-deficient or NLRP12-overexpressing cells. NLRP12 regulated the protein stability of HK2 but did not affect the transcriptional stability of HK2 mRNA (Figs. 6A, S2B). Because protein stability is regulated mainly by the ubiquitin–proteasome system and the autophagy system, we treated NLRP12-overexpression gastric cancer cells with MG132, a classical ubiquitination inhibitor, and CQ, an autophagy-lysosomal pathway inhibitor. The results indicated that NLRP12 may affect the protein degradation of HK2 through the lysosomal degradation pathway (Fig. 6B). Because the most prevalent autophagy-targeting signal in mammals is cargo K63-linked ubiquitination, we examined the level of HK2 ubiquitination in gastric cancer cells to further investigate whether the mechanism by which NLRP12 increases the expression of HK2 protein is related to ubiquitination [32]. After NRLP12 knockdown or overexpression, HK2 ubiquitination levels decreased or increased, respectively (Fig. 6C). Previous studies have indicated that the autophagy-regulated ubiquitination-mediated degradation of HK2 may be dependent mainly on K63-linked polyubiquitination; To directly interrogate the role of K63-linked ubiquitination, we transfected 293T cells with wild-type ubiquitin (Ub-K63) or a K63R ubiquitin mutant (Ub-K63R) that prevents K63-specific chain formation. NLRP12 suppresses K63-linked polyubiquitination of HK2, whereas expression of the Ub-K63R mutant abolishes this regulatory effect, confirming the specificity of NLRP12 toward K63-linked chains (Fig. 6D, E). In order to further clarify the level of HK2 protein in lysosomes, we performed lysosomal IP experiments. The experimental results demonstrate that HK2 can enter lysosomes. Following EBSS treatment, HK2 protein levels in lysosomes significantly increased, indicating that HK2 can be degraded through the lysosomal pathway. After NLRP12 knockdown, HK2 levels in lysosomes were significantly increased. Concurrently, we performed IF staining and quantitatively analyzed the colocalization of HK2 with lysosomes. Notably, NLRP12 knockdown significantly enhanced the colocalization between HK2 and lysosomes. These findings suggest that NLRP12 may suppress HK2 protein degradation by inhibiting its lysosomal targeting (Fig. 6F, G).

### NLRP12 suppresses TRIM25-mediated K63-linked ubiquitination of HK2 via competitive binding

**Fig. 7:**
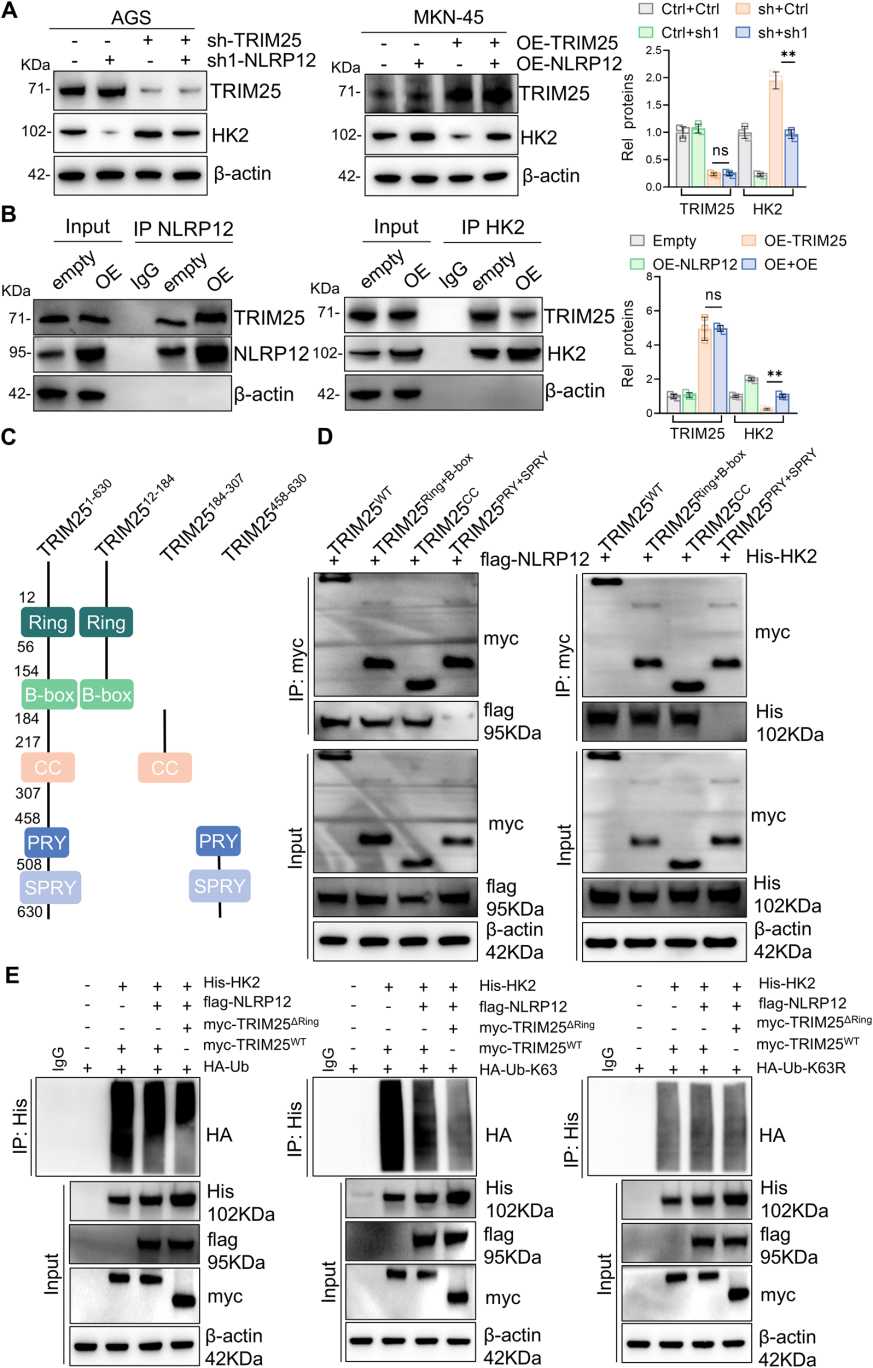
NLRP12 suppresses TRIM25-mediated K63-linked ubiquitination of HK2 via competitive binding. **A** TRIM25 and HK2 protein levels after TRIM25 knockdown or overexpression in NLRP12 knockdown cells were detected by Western blot analysis, and quantitative analysis was performed (*n* = 3, means ± SD, **P < 0.01). **B** The binding levels of TRIM25 and NLRP12 were detected by Co-IP. **C** Different TRIM25 truncations depicted in pattern diagram. **D** IP was used to analyze the expression levels of different TRIM25 truncations combined with flag-NLRP12 or His-HK2 in 293T cells. **E** 293T cells were transfected with His-HK2, flag-NLRP12, myc-TRIM25^ΔRing^, myc-TRIM25^WT^ and HA-Ub, HA-Ub-K63, HA-Ub-K63R. Cell lysates were subjected to IP with the indicated antibodies.

IP-MS screening identified an E3 ligase TRIM25 interacting with HK2. Unexpectedly, knockdown or overexpression of NLRP12 did not significantly alter the transcription or protein levels of TRIM25 (Fig. S3A, B). Previous studies have shown that NLRP12 can prevent TRIM25-mediated Lys63 ligase ubiquitination [24]. Further research has revealed that knockdown of TRIM25 significantly reversed the decrease in HK2 expression caused by knockdown of NLRP12, and overexpression of TRIM25 significantly reduced the increase in HK2 expression caused by overexpression of NLRP12 (Fig. 7A). Co-IP confirmed NLRP12-TRIM25 interaction, with increased NLRP12-TRIM25 binding upon NLRP12 overexpression and decreased TRIM25-HK2 binding when TRIM25 was overexpressed (Fig. 7B). Molecular docking and SPR analyses demonstrated direct binding between NLRP12 and TRIM25 (ΔG: −11.8 kcal/mol; KD: 3.64 × 10^−^⁷M) (Fig. S3C, D). TRIM25 acts as a classical ubiquitin E3 ligase. Systematic interrogation of polyubiquitin chain linkages (K6, K11, K27, K29, K33, K48, K63) revealed that TRIM25-mediated K63-linked ubiquitination of HK2 exhibited the most prominent enrichment (Fig. S3E). Furthermore, domain-specific truncation analysis of TRIM25 demonstrated that NLRP12 competes with HK2 for binding to its Ring/B-box/CC domain rather than PRY/SPRY domain. NLRP12 overexpression markedly reduced the interaction between TRIM25 and HK2, which consequently impaired TRIM25-dependent K63-linked ubiquitination of HK2 and its lysosomal degradation (Fig. 7C, D). Subsequently, we generated domain-deficient mutants (ΔRing) and performed ubiquitination assays. Both total ubiquitination and K63-linked polyubiquitination of HK2 were markedly diminished in TRIM25^ΔRing^-expressing cells. Strikingly, expression of a K63 linkage-defective ubiquitin mutant (Ub-K63R) abolished HK2 ubiquitination mediated by either TRIM25^WT^ or TRIM25^ΔRing^ to equivalent extents. These data establish that TRIM25 selectively drives K63-linked polyubiquitination of HK2 through its functional Ring domain (Fig. 7E). Notably, genetic perturbation of TRIM25 (knockdown or overexpression) effectively rescued the NLRP12-mediated modulation of proliferative capacity in gastric carcinoma cells (Fig. S3F). NLRP12 competitively antagonizes HK2 for TRIM25 binding, with upregulated NLRP12 expression effectively suppressing TRIM25-HK2 complex formation. This molecular competition impedes TRIM25-dependent K63-linked ubiquitination of HK2, thereby blocking its degradation and consequently promoting malignant proliferation in gastric cancer cells.

### NLRP12 promotes gastric cancer progression through HK2 in vivo

**Fig. 8:**
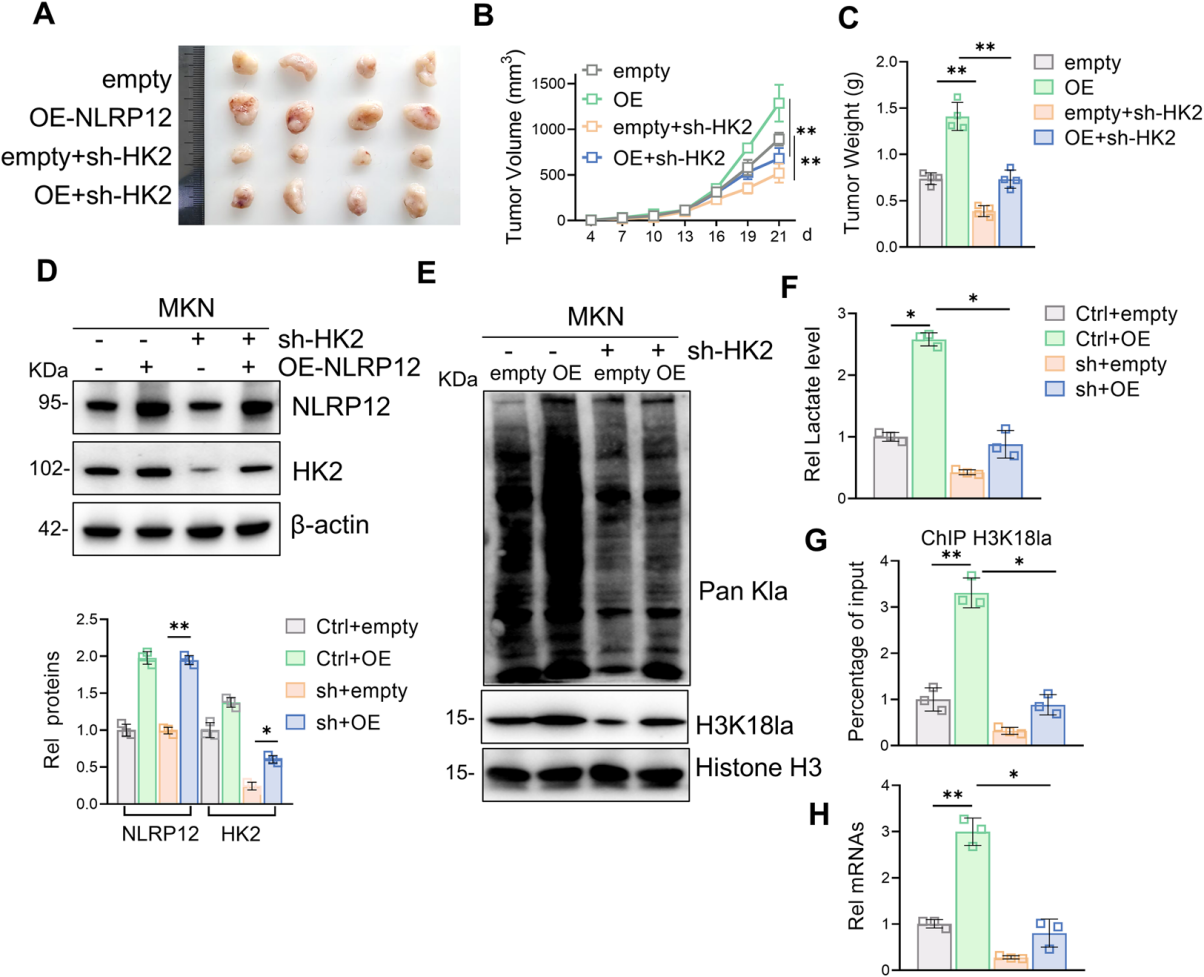
NLRP12 promotes HK2-mediated gastric cancer progression in vivo. **A** Control cells, OE-NLRP12 cells, sh-HK2 cells or OE-NLRP12+sh-HK2 cells were injected subcutaneously into the sides of BALB/c nude mice to observe the proliferation ability of the cells. Anatomical tumor images of BALB/c nude mice (*n* = 4). **B** Tumor volume was assessed (*n* = 4, means ± SD, **P < 0.01). **C** Tumor weights were assessed (*n* = 4, means ± SD, **P < 0.01). **D** Protein levels of NLRP12 and HK2 in tumor tissues were detected by Western blot analysis, and quantitative analysis was performed (*n* = 4, means ± SD, *P < 0.05 and **P < 0.01). **E** Histone lactylation and H3K18la levels in tumor tissues were detected by Western blot analysis. **F** Lactic acid levels in tumor tissues were detected (*n* = 4, means ± SD, *P < 0.05, **P < 0.01). **G** The level of H3K18la-bound Myc in tumor tissue was detected by a ChIP assay (*n* = 4, means ± SD, *P < 0.05). **H** The level of Myc transcription in tumor tissues was detected by RT-qPCR (*n* = 4, means ± SD, *P < 0.05 and **P < 0.01).

To evaluate the specific mechanism by which NLRP12 promotes gastric cancer in vivo, we established xenograft gastric cancer models. Compared with control treatment, NLRP12 overexpression significantly increased tumor burden parameters (size/volume/weight), whereas HK2 knockdown significantly decreased these tumorigenic metrics (Fig. 8A–C). In addition, analysis of tumor tissues revealed that NLRP12 promoted HK2 expression, histone lactylation, H3K18la, lactate levels and Myc transcription in vivo, all of which were reversed by sh-HK2 intervention (Fig. 8D–H). These findings establish that NLRP12 drives gastric carcinogenesis in vivo through HK2-dependent regulation of H3K18la modification and Myc signaling.

## Discussion

**Fig. 9:**
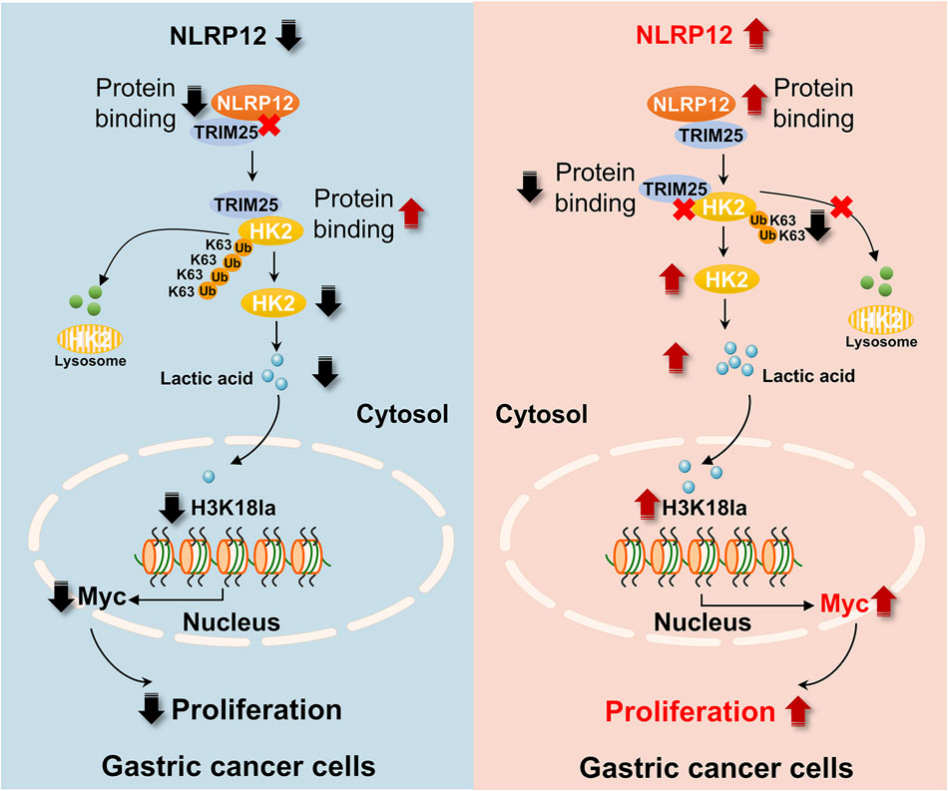
Mechanism by which NLRP12 promotes the malignant progression of gastric cancer. Within gastric cancer cells, NLRP12 binds to TRIM25. This binding prevents TRIM25 from participating in the K63-linked chains of HK2. Consequently, this prevents HK2 from undergoing degradation via the autophagic-lysosomal pathway. This blockade enhances the protein stability of HK2, thereby upregulating the expression of HK2 and promoting glycolysis in gastric cancer cells. The high expression of HK2 promotes the production and accumulation of lactate, leading to H3K18 lactylation (H3K18la) in gastric cancer cells. H3K18la promotes the transcription of Myc, ultimately promoting the progression of gastric cancer. The image was created via BioRender.com.

As inflammasomes, the NLR family was initially thought to regulate various inflammatory pathways, but it has recently been shown to play a key role in tumors [33]. Inflammasomes are involved in the regulation of tumorigenic signaling pathways in various tumor cells and can serve as effective therapeutic targets [34]. The present study revealed that NLRP12 is highly expressed in gastric cancer and promotes gastric cancer development, and this ability is due mainly to its ability to promote glucose metabolism and lactic acid metabolism in gastric cancer cells. Mechanistically, NLRP12 binds to TRIM25 to prevent TRIM25 from participating in the K63-linked ubiquitination of HK2, which subsequently enhances the protein stability of HK2, thereby upregulating the expression of HK2 and promoting glycolysis in gastric cancer cells. High expression of HK2 promotes the production and accumulation of lactic acid, resulting in the lactylation of H3K18 in gastric cancer cells. H3K18la promotes the transcription of Myc, ultimately promoting the progression of gastric cancer (Fig. 9). We investigated the mechanism of NLRP12 in gastric cancer and revealed the specific pathway regulating metabolic reprogramming, which may provide new targets and new ideas for gene therapy in gastric cancer.

Metabolic reprogramming is now recognized as a signature change in malignant tumors [35]. Gastric cancer cells have strong glycolytic and lactic acid production abilities, which continuously provide essential substances and energy for the malignant proliferation of gastric cancer cells, and these abilities increase with increasing degree of malignancy in gastric cancer [36]. Therefore, the inhibition of glycolysis or lactic acid production to inhibit the progression of gastric cancer is an effective treatment. Previous studies have reported that N4-acetylcytidine drives glycolysis in gastric cancer via a NAT10/SEPT9/HIF-1α positive feedback loop [37], and the lncRNA CCAT1 facilitates the progression of gastric cancer via PTBP1-mediated enhancement of glycolysis [38]. In addition, lactic acid, a glycolysis product, can be used to predict the malignant progression and immune escape of gastric cancer [5], and we believe that glycolysis- and lactylation-related genes can be used as potential therapeutic targets for diagnostic markers of gastric cancer. The present results are consistent with those of previous studies, suggesting that the inhibition of glycolysis and histone lactylation are effective strategies for the treatment of gastric cancer.

An important finding of the present study was that abnormally elevated NLRP12 expression during the progression of gastric cancer is strongly associated with the regulation of abnormal proliferation of gastric cancer cells through the ubiquitination of HK2 and subsequent HK2-mediated H3K18la. Therefore, HK2 inhibition is crucial for exploring targeted therapies for gastric cancer [39]. Currently, the small-molecule compound 2-DG is widely used to inhibit HK2 and glycolysis, but its specific mechanism of action and safety still need to be further studied [40]. Therefore, the regulation of HK2 still needs to be further explored. There are mainly two protein degradation pathways, including ubiquitin‒proteasome and autophagolysosomal modifications [41]. As a key molecule in glycolysis, HK2 undergoes ubiquitination modification. For example, TRIM36 inhibits glycolysis through Lys48-mediated HK2 ubiquitination [18]. Degradation of HK2 by chaperone-mediated autophagy promotes metabolic catastrophe and cell death [42]. The present study revealed that overexpressed NLRP12 binds to TRIM25, which prevents K63-linked ubiquitination of HK2, thereby preventing HK2 degradation. Moreover, HK2 overexpression significantly reversed the inhibitory effect on the proliferation of gastric cancer cells induced by NLRP12 knockdown. Because the mechanisms by which ubiquitination modifications affect specific gene expression are interrelated, the present study not only reveals important protein degradation mechanisms for HK2 inhibition and glycolysis but also suggests that the anti-gastric cancer effects of HK2 inhibition and metabolic heterogeneity can be achieved through NLRP12 and other possible molecular interventions.

Interestingly, the present study revealed that NLRP12 is involved in regulating the ubiquitination-mediated degradation of HK2 through the binding of TRIM25. Previous studies have shown that TRIM25 promotes the progression of gastric cancer. For example, TRIM25 promotes SP1 ubiquitination at K610, further inhibiting the expression of MMP2 and angiogenesis in gastric cancer [43]. In the present study, TRIM25 expression did not change after NLRP12 knockdown; therefore, we confirmed that NLRP12 competes with HK2 for binding the Ring/B-box/CC domain rather than the PRY/SPRY domain of TRIM25. NLRP12 has been widely verified as an inflammatory regulator, but its role in regulating gastric cancer has not been confirmed. The present study explored the mechanism of NLRP12 in depth, broadening the scope and ideas for the regulation of cancer by the NLR family.

The present study had several limitations. For example, it lacked further mechanistic validation using in situ models of gastric cancer. Will NLRP12 affect other types of post-translational modifications of HK2? In addition, the development of chemotherapy resistance is a serious problem in the clinical treatment of gastric cancer, and it remains unknown whether NLRP12 can reduce chemotherapy resistance in gastric cancer.

In summary, the present study revealed that NLRP12-mediated HK2 ubiquitination is the main mechanism to regulate glycolysis and histone lactylation in gastric cancer and is important for the progression of this disease. NLRP12, as an inflammatory regulatory gene, is mainly studied due to its immunomodulatory function. This study is the first to show that NLRP12 in gastric cancer cells promotes gastric cancer progression through metabolic reprogramming. Because glycolysis and histone lactylation are reversible and their inhibitors are emerging as effective clinical options, the present findings provide strong evidence for clinical therapeutic applications for gastric cancer.

## Supplementary information

Supplementary figures

original image

## Data Availability

All original data reported in this paper are available from the corresponding author upon request.
