# Supplementary material for: NLRP12 decreases TRIM25-mediated HK2 degradation to promote glycolysis and H3K18la in gastric cancer
Source: Cell Death Dis. 2025 Aug 13;16(1):615. doi: 10.1038/s41419-025-07923-3 (PMC12343871; doi:10.1038/s41419-025-07923-3)
Supplement: Supplementary file 1 — Supplementary figures [file 41419_2025_7923_MOESM1_ESM.pdf]

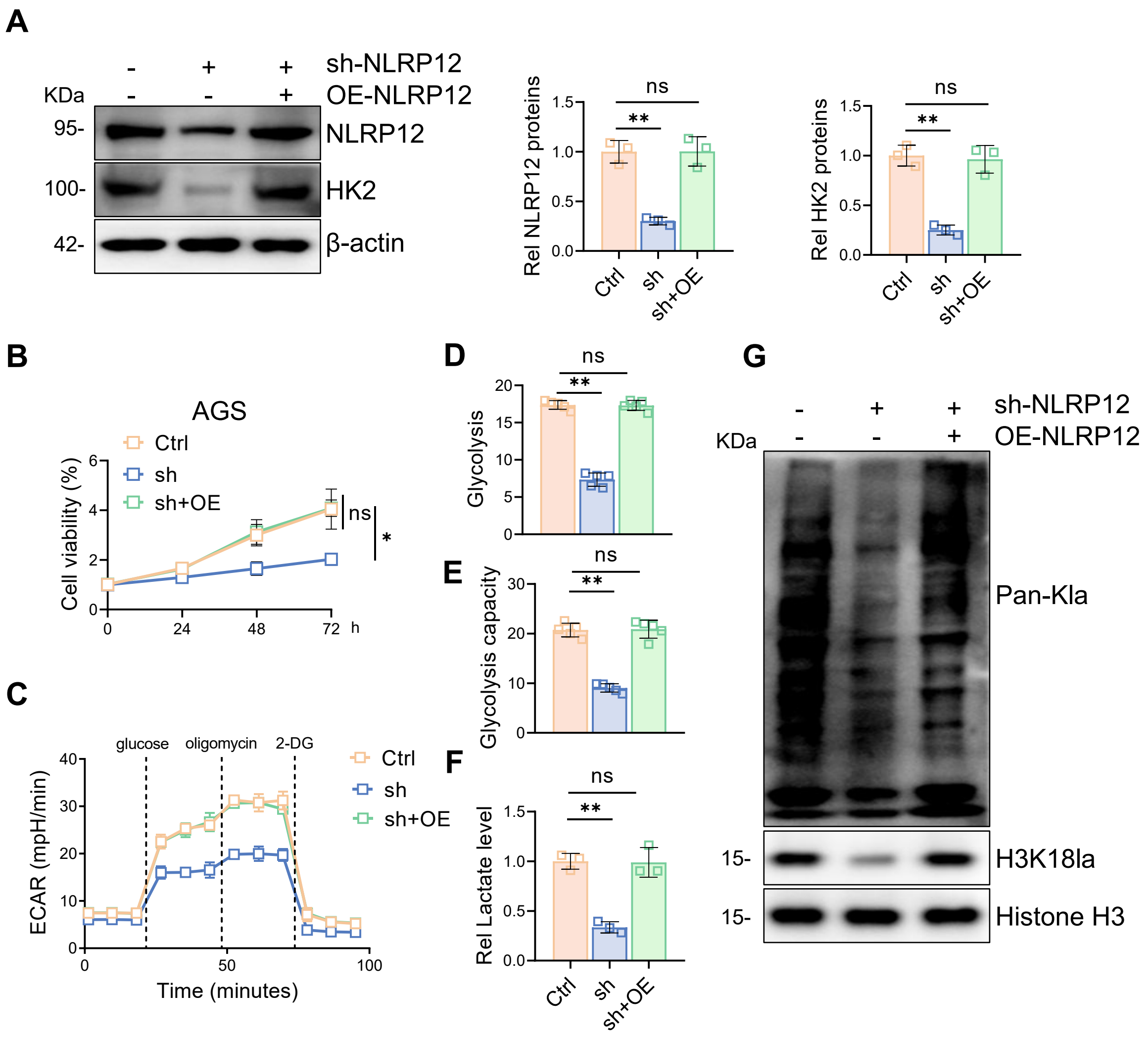

Figure.S1: NLRP12 promotes the malignant progression of gastric cancer cells. **A** Western blot analysis was used to detect NLRP12 and HK2 protein expression levels, which overexpressed NLRP12 after knockdown NLRP12. **B** A CCK-8 assay was used to evaluate the changes in gastric cancer cell viability, which overexpressed NLRP12 after knockdown NLRP12. **C** A Seahorse assay was used to evaluate the changes in the extracellular acidification rate of gastric cancer cells, which overexpressed NLRP12 after knockdown NLRP12. **D** The changes in glycolysis in gastric cancer cells, which overexpressed NLRP12 after knockdown NLRP12. **E** The changes in the glycolytic capacity of gastric cancer cells, which overexpressed NLRP12 after knockdown NLRP12. **F** The changes in lactate levels in gastric cancer cells, which overexpressed NLRP12 after knockdown NLRP12. **G** Histone lactylation and H3K18la expression in gastric cancer cells, which overexpressed NLRP12 after knockdown NLRP12.

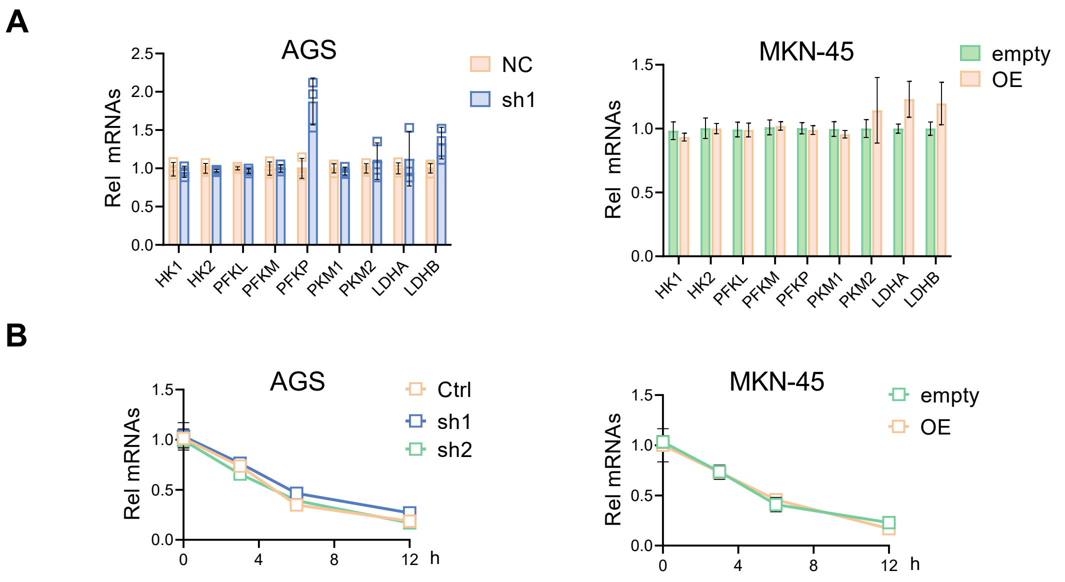

Figure.S2: NLRP12 does not affect the transcriptional level and mRNA stability of HK2. **A** Transcriptional levels of glycolytic enzymes were detected by RT-qPCR after the knockdown or overexpression of NLRP12 (n=3, means ± SDs). **B** The mRNA stability of HK2 was detected after Actd treatment and after knockdown or overexpression of NLRP12 (n=3, means ± SDs).

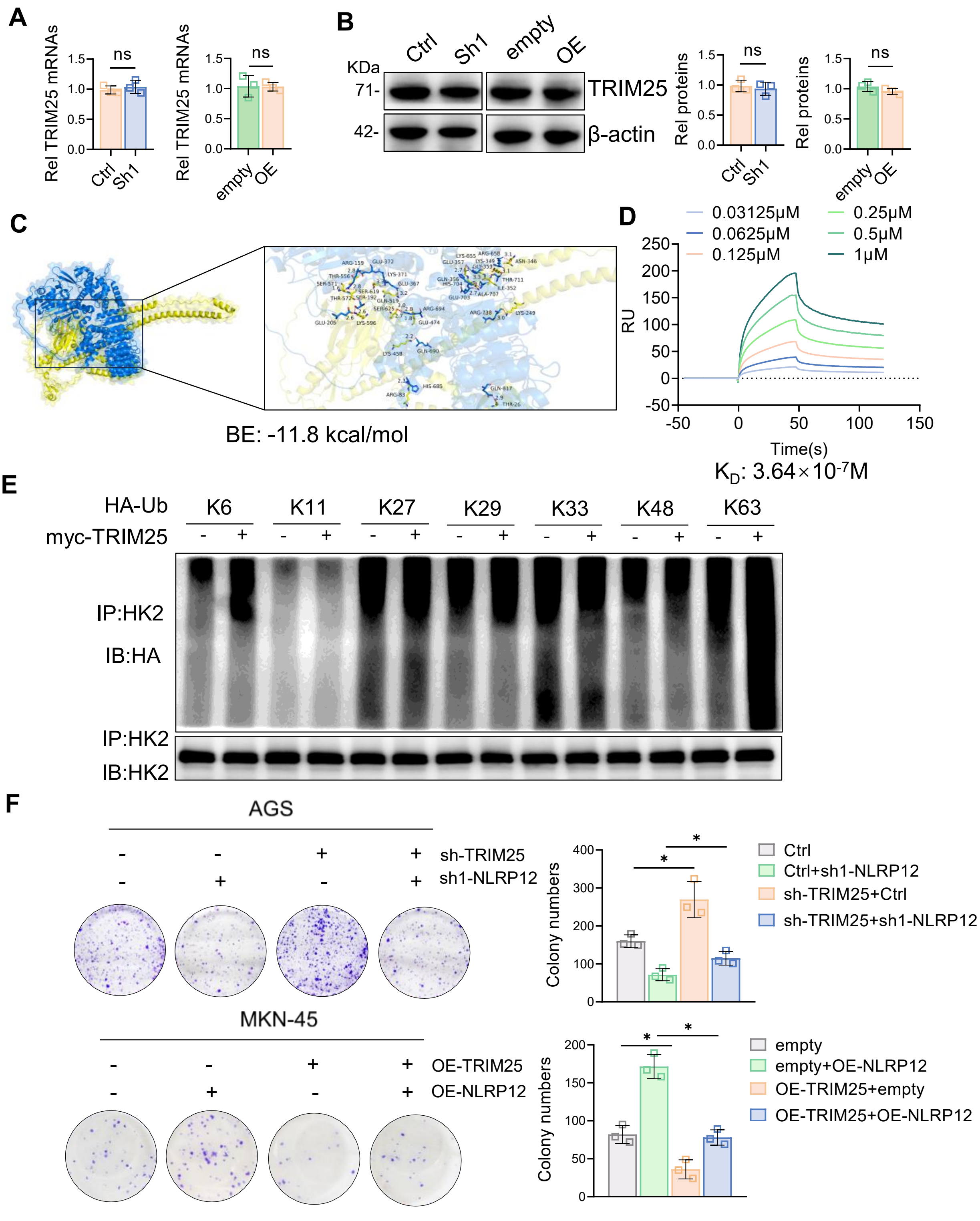

Figure.S3: NLRP12 combined with TRIM25 affects Ub-K63 of HK2 and promotes the malignant progression of gastric cancer. **A** The mRNA level of TRIM25 after NLRP12 knockdown was detected by RT-qPCR (n=3, means ± SD, *$P < 0.05$). **B** The protein level of TRIM25 after the knockdown of NLRP12 was detected by Western blot analysis, and quantitative analysis was performed (n=3, means ± SDs). **C** Molecular docking analysis of NLRP12 binding to TRIM25 with binding energies of -11.8 kcal/mol. **D** Surface Plasmon Resonance analysis of NLRP12 binding to TRIM25 with K_D: 3.64×10^{-7}M. **E** 293T cells were transfected with myc-TRIM25 and HA-Ub-K6, K11, K27, K29, K33, K48, K63. Cell lysates were subjected to IP and IB with the indicated antibodies. **F** The proliferation of gastric cancer cells after the knockdown or overexpression of TRIM25 and NLRP12 was examined by a colony formation assay.
