# Supplementary figures and images for: NLRP12 decreases TRIM25-mediated HK2 degradation to promote glycolysis and H3K18la in gastric cancer
Source: Cell Death Dis. 2025 Aug 13;16(1):615. doi: 10.1038/s41419-025-07923-3 (PMC12343871; doi:10.1038/s41419-025-07923-3)

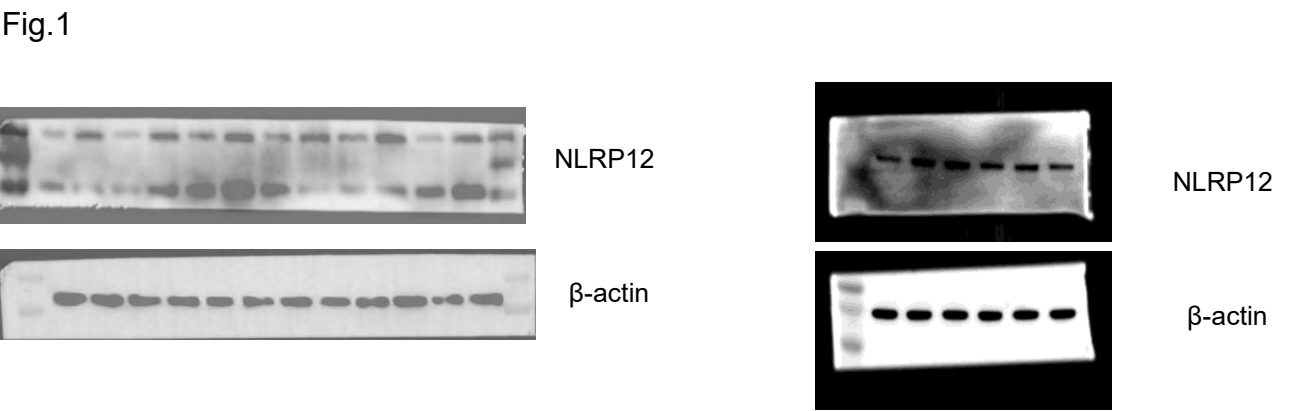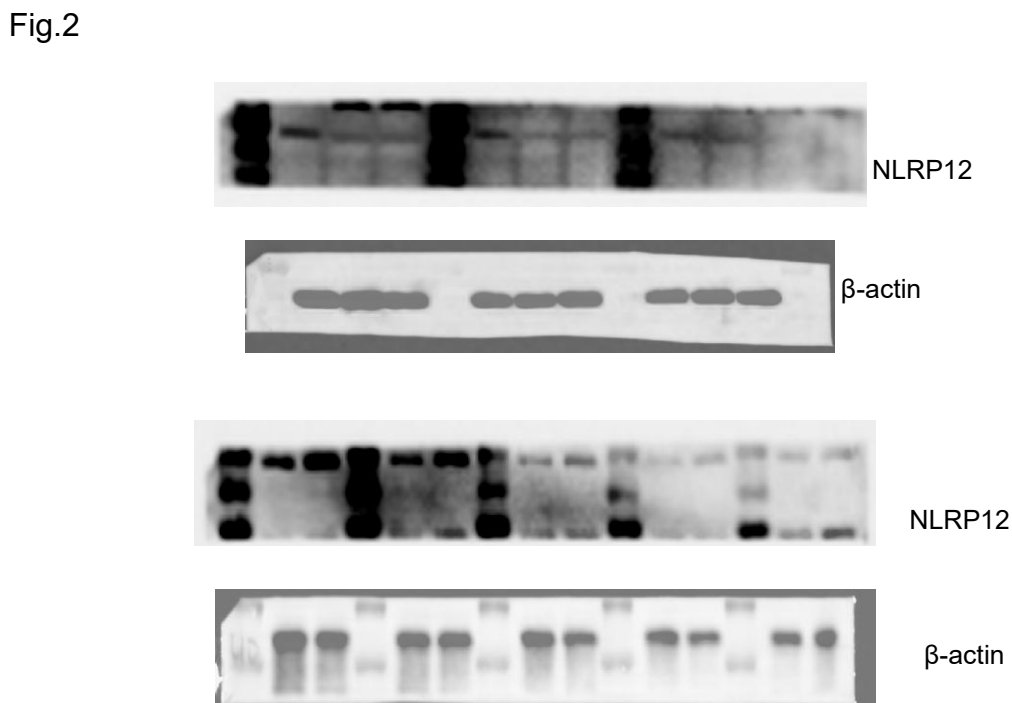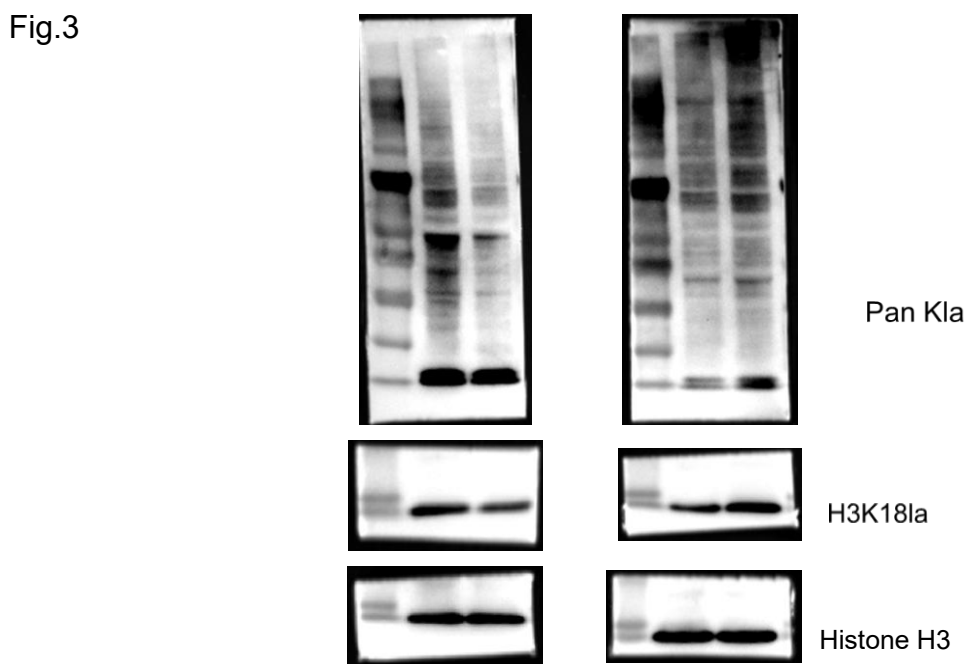

Fig.4

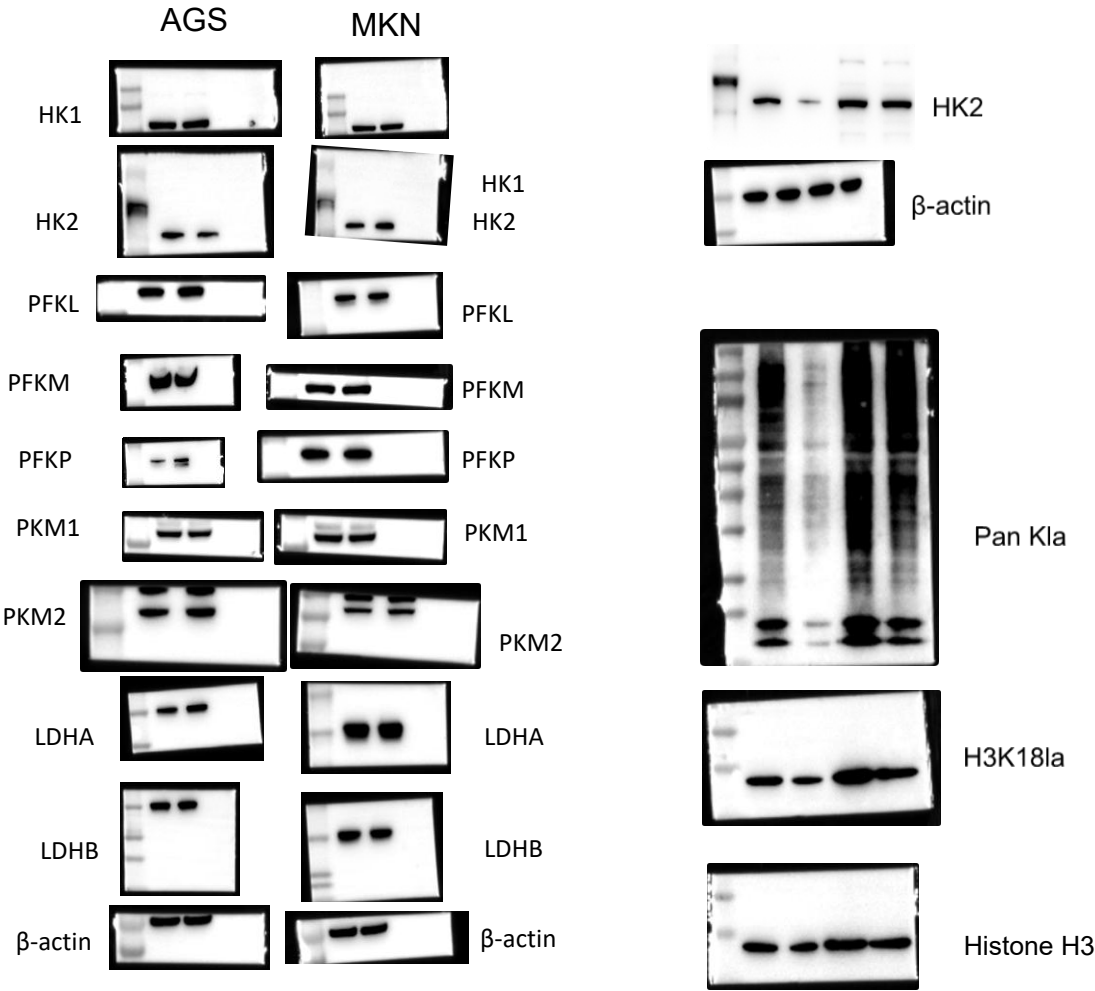

Fig.5

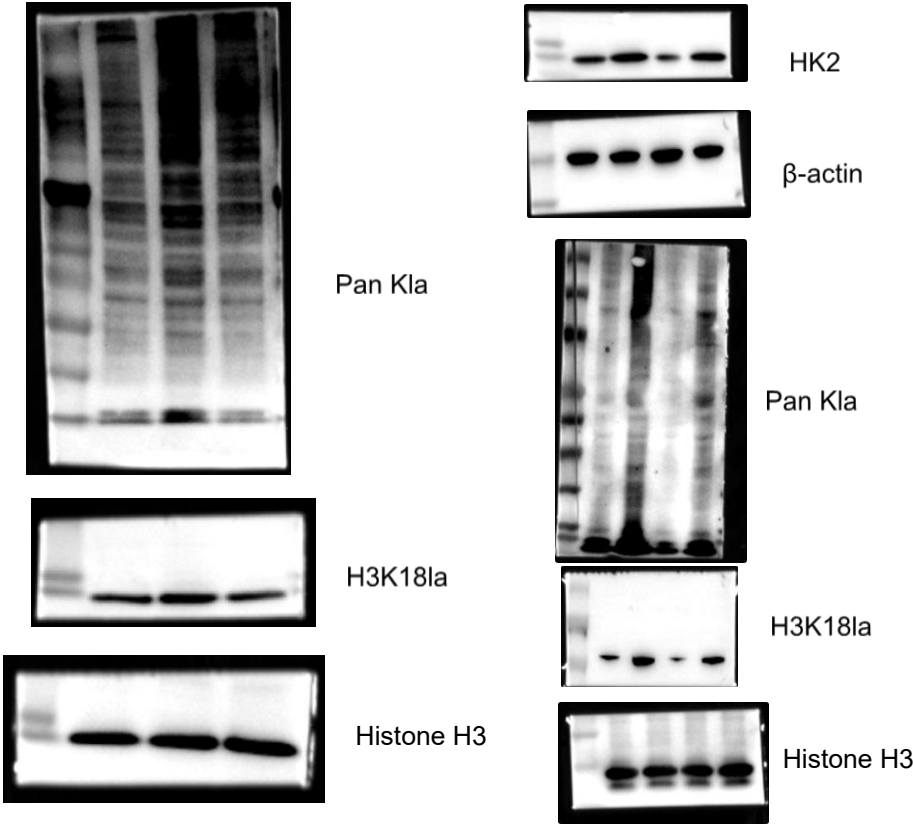

Fig.6

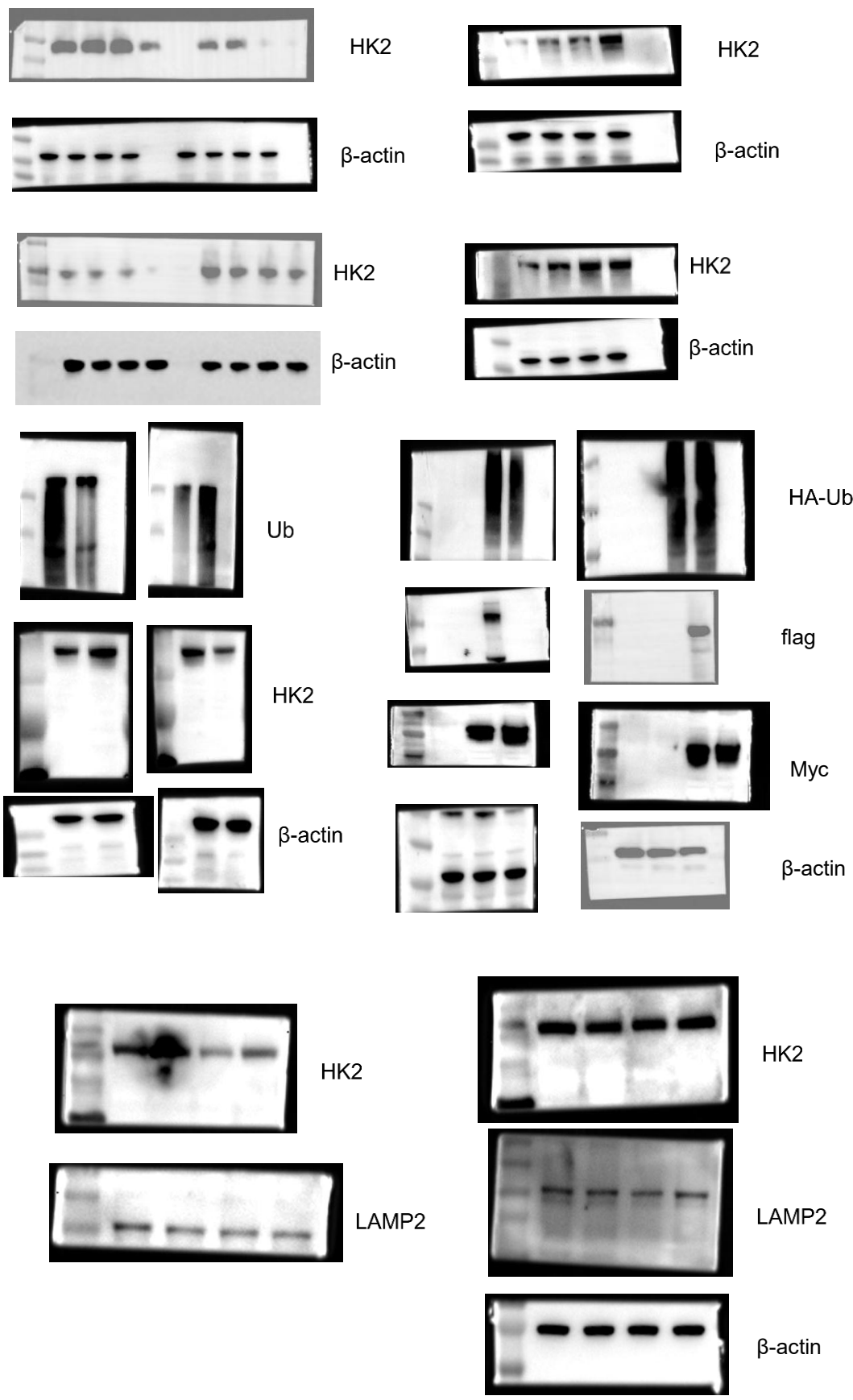

Fig.7

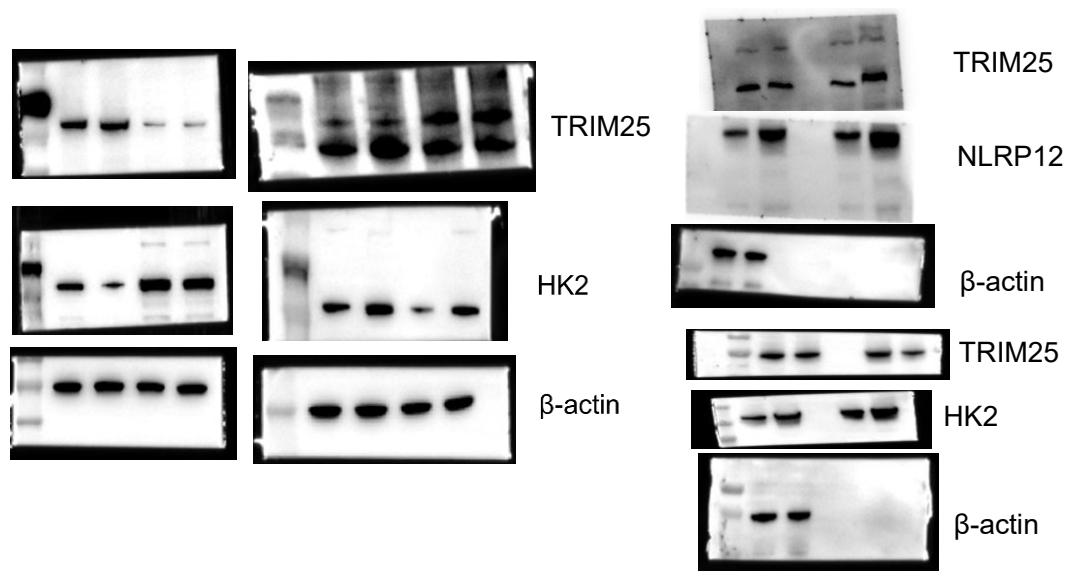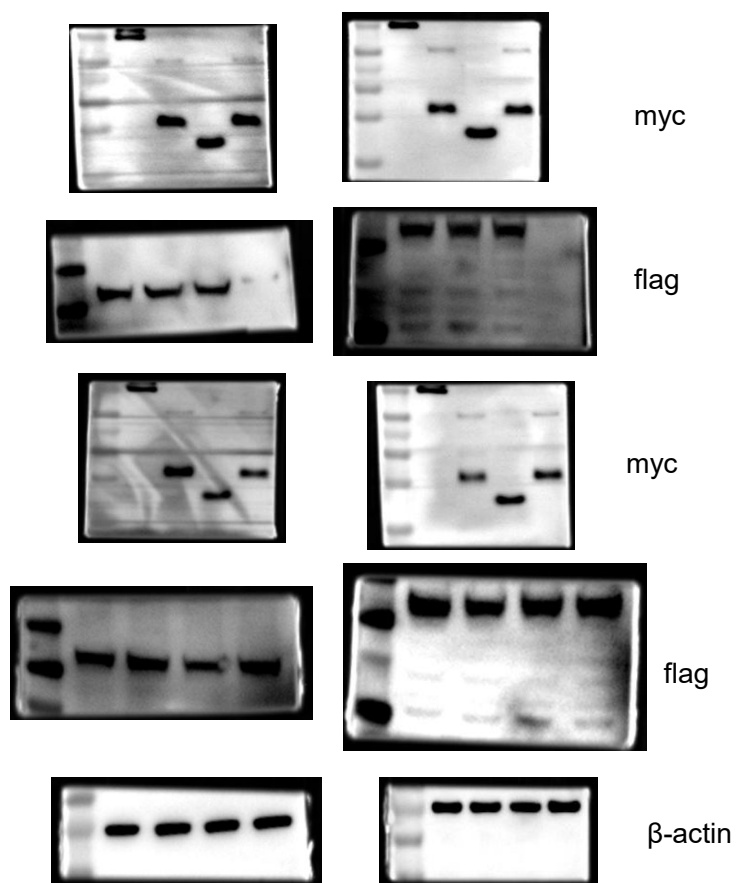

Fig.7

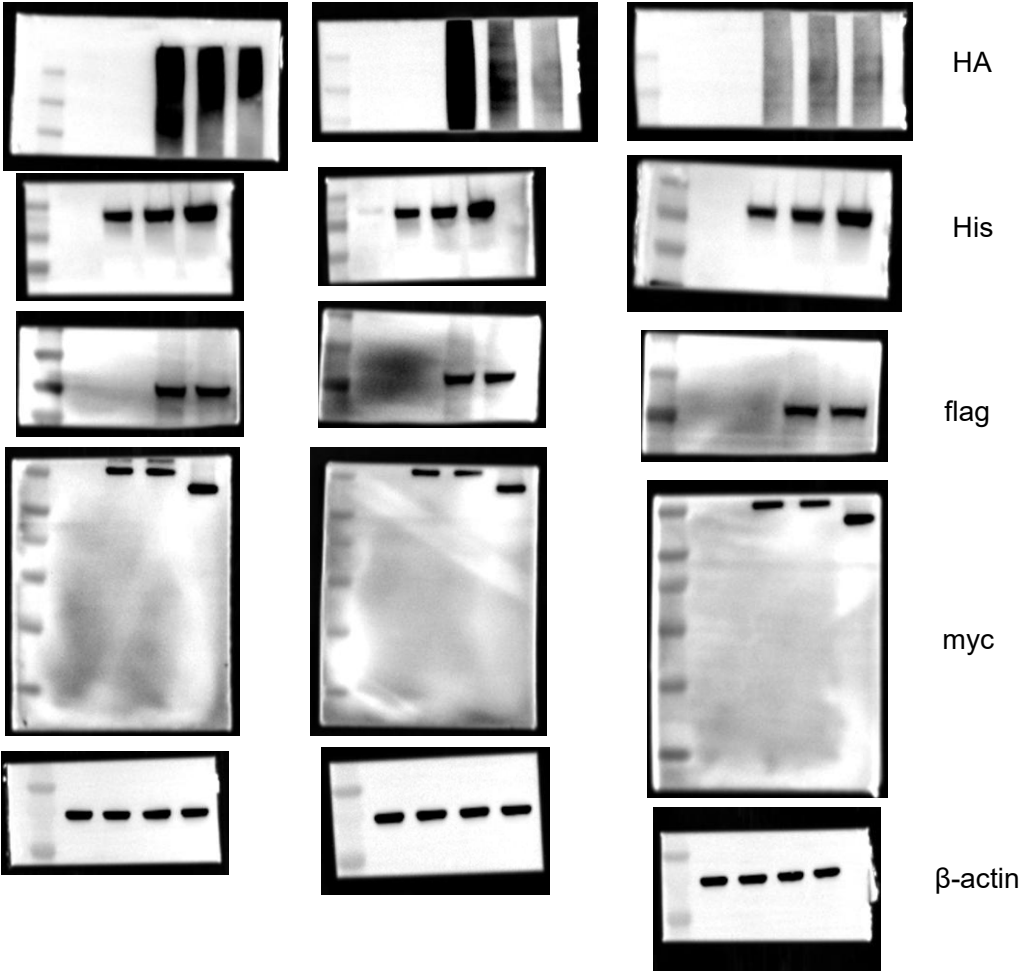

Fig.8

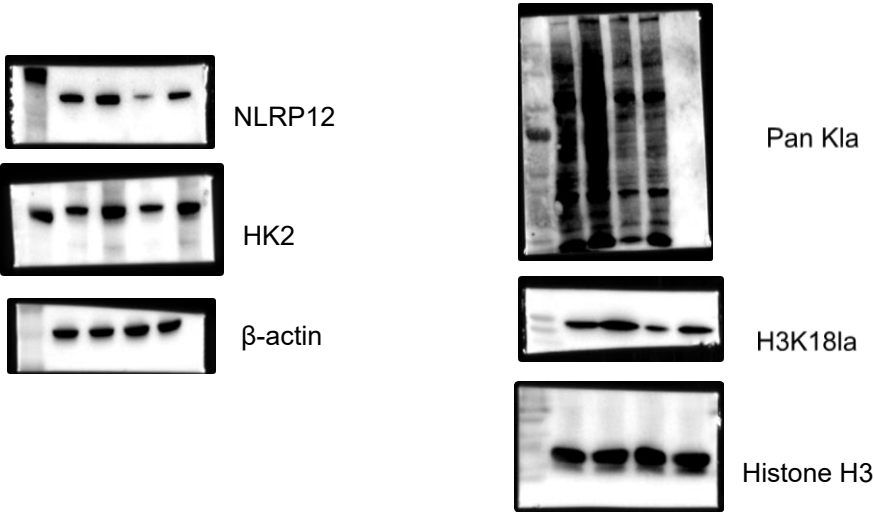

Fig.S1

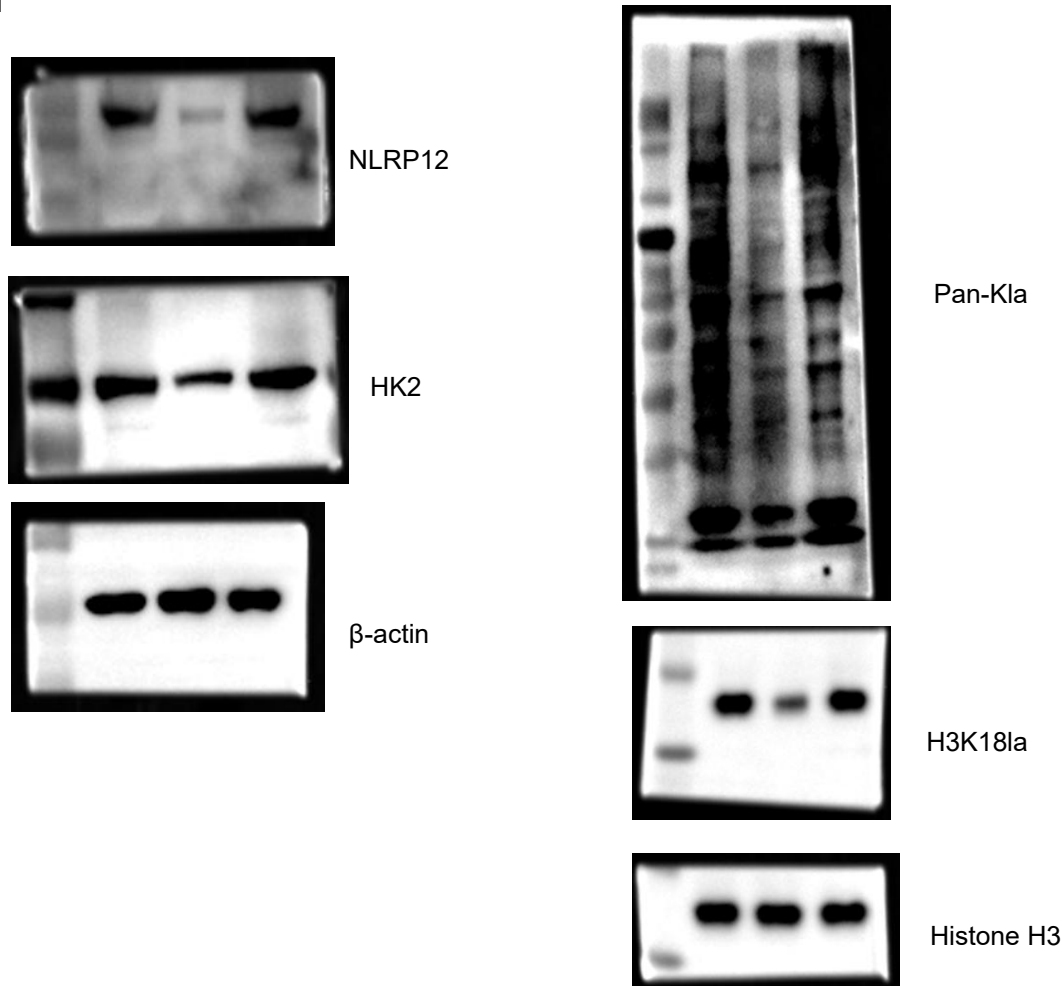

Fig.S3

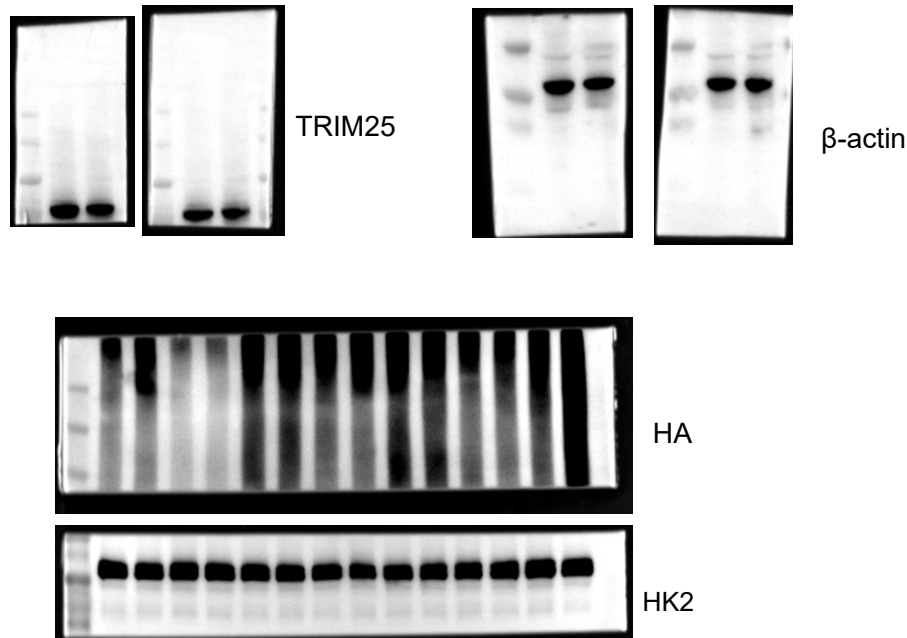

Supplement: Supplementary file 2 — original image [file 41419_2025_7923_MOESM2_ESM.pdf]
